# The Effects of Hormonal Contraceptives on the Brain: A Systematic Review of Neuroimaging Studies

**DOI:** 10.3389/fpsyg.2020.556577

**Published:** 2020-10-27

**Authors:** Marita Kallesten Brønnick, Inger Økland, Christian Graugaard, Kolbjørn Kallesten Brønnick

**Affiliations:** ^1^Center for Clinical Research in Psychosis (TIPS), Stavanger University Hospital, Stavanger, Norway; ^2^Department of Clinical Medicine, Center for Sexology Research, Aalborg University, Aalborg, Denmark; ^3^Department of Obstetrics and Gynecology, Stavanger University Hospital, Stavanger, Norway; ^4^Department for Caring and Ethics, Faculty of Health Sciences, University of Stavanger, Stavanger, Norway; ^5^SESAM, Department of Psychiatry, Stavanger University Hospital, Stavanger, Norway; ^6^Department of Public Health, Faculty of Health Sciences, University of Stavanger, Stavanger, Norway

**Keywords:** hormonal contraceptives, brain, neuroimaging, MRI, PET, EEG

## Abstract

**Background:** Hormonal contraceptive drugs are being used by adult and adolescent women all over the world. Convergent evidence from animal research indicates that contraceptive substances can alter both structure and function of the brain, yet such effects are not part of the public discourse or clinical decision-making concerning these drugs. We thus conducted a systematic review of the neuroimaging literature to assess the current evidence of hormonal contraceptive influence on the human brain.

**Methods:** The review was registered in PROSPERO and conducted in accordance with the PRISMA criteria for systematic reviews. Structural and functional neuroimaging studies concerning the use of hormonal contraceptives, indexed in Embase, PubMed and/or PsycINFO until February 2020 were included, following a comprehensive and systematic search based on predetermined selection criteria.

**Results:** A total of 33 articles met the inclusion criteria. Ten of these were structural studies, while 23 were functional investigations. Only one study investigated effects on an adolescent sample. The quality of the articles varied as many had methodological challenges as well as partially unfounded theoretical claims. However, most of the included neuroimaging studies found functional and/or structural brain changes associated with the use of hormonal contraceptives.

**Conclusion:** The included studies identified structural and functional changes in areas involved in affective and cognitive processing, such as the amygdala, hippocampus, prefrontal cortex and cingulate gyrus. However, only one study reported primary research on a purely adolescent sample. Thus, there is a need for further investigation of the implications of these findings, especially with regard to adolescent girls.

## Introduction

Synthetic sex hormones became available as contraceptive drugs in the 1960's, and they are currently being used by more than 100 million women worldwide (Christin-Maitre, 2013). In the US, it is estimated that 88% of all women of fertile age have utilized this type of birth control at some point in their lives (Daniels and Jones, 2013). Sex hormones consist of androgens, estrogens and progesterone, and *in vivo* they are synthesized in the gonads, the adrenal glands and the brain. They profoundly impact the brain *during fetal life*, exerting epigenetic effects and directing development along male or female trajectories by influencing a variety of molecular and cellular processes. Moreover, they affect regional gray matter volumes and neural connectivity associated with psychosexual and other behavioral functions (Hines, 2006; Josso, 2008; Peper et al., 2011; McCarthy and Nugent, 2015).

Converging lines of evidence from animal literature, as well as cognitive and affective neuroscience involving human subjects, suggest that these hormones *continue to shape the brain postnatally*, also during adolescence (Herting et al., 2014; Schulz and Sisk, 2016). In adulthood, they modulate brain areas involved in cognitive and emotional processing, and they are implicated in mood and anxiety disorders (Comasco et al., 2014; Toffoletto et al., 2014; Garcia et al., 2018). If the synthetic sex hormones contained within hormonal contraceptives (HC) (Christin-Maitre, 2013) interact with sex hormone receptors in the brain, they have the potential to interfere with multiple neurohormonal regulatory mechanisms and neural structures involved in emotion, cognition and psychosexual behavior (Fuhrmann et al., 2015; Schulz and Sisk, 2016). To date, neuroimaging research on the effects of HC use on the structure and function of the brain has not been systematically reviewed. The potential for influencing brain plasticity and hence altering brain structures and behavioral outcomes has therefore not been fully elucidated.

Plasticity represents an intrinsic ability of the nervous system to adapt its structure and function in response to endogenous and exogenous environmental demands. This ability persists throughout life (Pascual-Leone et al., 2005). However, there are periods of life when the brain exhibits an increased degree of plasticity and is particularly vulnerable to environmental changes. The perinatal phase is such a period. In 1959, Phoenix et al. proposed that perinatal sex hormones exert an organizing effect on the brain, with ensuing consequences for behavior (Phoenix et al., 1959). They found that prenatal exposure of female guinea pigs to testosterone masculinized their later mating behavior, and they went on to demonstrate similar findings in female rhesus monkeys, who displayed masculinized play patterns following prenatal testosterone treatment. Their claim was that, perinatally, testosterone has an *organizing* effect on the brain, while the hormonal events of puberty have an *activating/deactivating* effect on the anatomical structures previously organized.

Several researchers have since expanded on, and in part refuted, this theory. Schulz and Sisk presented evidence from animal studies suggesting that sex hormones may have an organizing effect on the brain long after birth, gradually declining and ending approximately at the resolution of puberty (Schulz and Sisk, 2016). Beltz and Berenbaum (2013) provided further support for the theory of continued ability of sex hormones to exert permanent effects in humans by showing that early puberty, and thus early exposure to adult-levels of sex hormones, in men was associated with better performance in a mental rotation task (Wai et al., 2010). Consequently, adolescence might also be a period sensitive to organizing effects of sex hormones; and the effects may be stronger, the younger the individual is when exposed.

During adolescence, several brain areas, in particular the prefrontal cortex (PFC), undergo extensive structural maturation through processes such as synaptic pruning, reorganization and myelination (Petanjek et al., 2011; Blakemore, 2012). The brain's functional architecture also undergoes maturational processes of optimizing connectivity in functional networks (Sherman et al., 2014). This prolonged developmental shaping and reorganization of neural circuits has implications for understanding the vulnerability of the brain during this period, as the plastic brain is the platform for learning and developing as well as for psychopathology and cerebral disease.

While *endogenous* sex hormones have well-documented effects on the brain, the influence of their synthetic counterparts, progestins and ethinylestradiol, which are most commonly used in oral contraceptive pills (Christin-Maitre, 2013), has been less extensively explored. However, there is reason to believe that also synthetic sex hormones could have a significant neural impact, particularly if taken when the young female brain is developing into its adult form. Behavioral effects of HC have been shown in cognitive tasks such as mental rotation and verbal expressional fluency (Beltz et al., 2015; Griksiene et al., 2018), and of more serious concern is the demonstrated association between these drugs and various affective adversities. Thus, Skovlund et al. conducted a large national cohort study in Denmark, where they collected and compared data from the National Prescription Register and the Psychiatric Central Research Register. They found a correlation between the use of HC and a subsequent first diagnosis of depression and the use of antidepressants. The increased risk of these adverse outcomes was noted to be the highest in adolescent women (Skovlund et al., 2016). The Skovlund group also investigated associations between HC intake and suicidal behavior and they found an increased risk for both attempted and committed suicide. Again, the increased risk was highest in adolescent women, and it peaked within 2 months of intake debut (Skovlund et al., 2018).

In order to assess the prevalence of HC use among Norwegian adolescents, we queried the Norwegian Prescription Database regarding usage of drugs (Norwegian Prescription Database, 2019) according to the Anatomical Therapeutic Chemical (ATC) code G03A (Hormonal contraceptives for systemic use). This database provides data on these drugs from 2004 to 2018, and it is possible to query separately for age groups such as 10–14 and 15–19. The usage for girls between the ages of 10 and 14 has more than doubled from 2004 to 2018, and in 2018 about 1.2 percent of all 10–14-year-old girls used some form of systemic HC. The numbers for girls between the ages of 15 and 19 have been quite stable at about 40 percent throughout the same period. Thus, a substantial proportion of young girls use these drugs and the usage has increased rapidly among the youngest adolescent girls in Norway.

The central aim of this review was to identify and critically appraise all peer-reviewed empirical studies published in English concerning human subjects that have investigated the effects of HC on brain structure and function through digital neuroimaging techniques, such as magnetic resonance imaging (MRI) and functional MRI (fMRI), as well as positron emission tomography (PET), electroencephalography (EEG) and magnetoencephalography (MEG).

Our main hypotheses were that HC use affects both brain structure and function in humans, and that there are effects on brain structures known to differ statistically in men and women, such as the PFC, hypothalamus, amygdala and hippocampus (Cahill, 2006), as well as on brain structures involved in visuospatial and verbal cognition. Additionally, we hypothesized that HC use have the most pronounced effects on brain structures if used during early adolescence.

## Methods

This review was conducted in accordance with the Preferred Reporting Items for Systematic and Meta-Analyses (PRISMA) guidelines (Moher et al., 2009), and it was registered in the PROSPERO International Prospective Register of Systematic Reviews (Registration number: CRD42019142427).

### Literature Search

Studies employing neuroimaging techniques to measure possible HC effects on either brain structure or function were considered. In order to be included, the studies should (a) be primary empirical studies, (b) be conducted on women of fertile age using HC, and (c) have either a separate control group of naturally cycling (NC) women of comparable age or have HC users constitute their own controls by performing repeated assessments under NC and HC conditions. Thus, case reports, literature reviews and experimental studies with no control group were excluded. We included articles published in English from 1990 and up until February 2020. Studies older than the 90's are based on imaging techniques not comparable to those of modern neuroimaging.

### Stage One Search

The review was carried out in two stages. The first stage consisted of an exploratory search using PubMed and Google Scholar. PubMed covers most studies involving neuroscience and related fields, and Google Scholar indexes most broadly of all peer-review databases. We first combined the keyword “contraceptives” with “brain,” “cognition,” “emotion,” and “motivation” and searched the databases. We selected and read relevant review articles. The knowledge gained from this process was used to decide on keywords for the stage two searches.

### Stage Two Search

Following the initial exploratory search, systematic searches were carried out, employing a two-pronged approach aiming to identify structural and functional neuroimaging studies separately. In Table 1, the PICOS criteria for the searches are described.

**Table 1:** PICOS search.

P (Patient, problem, or population)	Women of fertile age using hormonal contraceptives
I (Intervention)	Hormonal contraceptives for women
C (Comparison, control, or comparator)	Currently and/or ever non-users of hormonal contraceptives of comparable age and health status or repeated assessment after start of use with pre-use as baseline.
O [Outcome(s)]	Functional or structural brain imaging measures
S (Study type)	Randomized controlled trials, quasi-experiments or observational studies (cohort or case-control)

We first combined search terms such as “contraceptive agent” and “birth control” with terms descriptive of *structural* neuroimaging such as “magnetic resonance imaging,” “computed axial tomography,” and “diffusion tensor imaging.” We searched for these terms in titles, abstracts and keywords as well as MESH- and Emtree-terms. Titles and abstracts were scanned, excluding articles not meeting our inclusion criteria. Finally, full texts were read in order to identify measures and methodological detail, further excluding ineligible articles. See Appendix 1 for a comprehensive list of search terms.

The second systematic search was carried out using the same search terms describing HC, this time combining them with terms aiming to identify *functional* neuroimaging studies. Relevant terms were “functional magnetic resonance imaging,” “positron emission tomography,” “electroencephalography,” and “event related potentials.” The same procedures of selection were carried out and relevant articles were retrieved. The stage 2 searches were carried out in February 2020.

A final reference and citation search strategy was employed to ensure that all relevant studies were identified. This implied scanning reference lists in the included articles as well as articles that cited the included papers, after a consensus selection process as described below.

After completing the systematic searches, the authors MKB and KKB independently read the keywords, abstracts and titles and divided articles into “included,” “excluded,” and “undecided” categories. After the initial assessments, full texts were read, and the researchers discussed the criteria and revised the “undecided” articles until all citations were either included or excluded.

Quality assessment was not done using a rigid framework resulting in a single numeric score, as the studies differed regarding dependent variables and design. However, we applied the validity typology of Donald Campbell and Thomas D. Cook (Cook et al., 1979) in order to assess threats to construct, internal, external and statistical conclusion validity. This was done as the study designs and outcome measures were heterogenous, necessitating a flexible approach for quality assessment. These dimensions of validity encompass most of the common causes of bias and validity threats regarding causal inference. Three levels of validity were applied: low, intermediate and high. Low validity implies that there was a validity threat serious enough to fundamentally invalidate the study. Intermediate implies that there were validity threats, but that they were outweighed or resolved to a degree that they were unlikely to seriously bias or confound the study. High means that there were no validity threats for the dimension in question. The assessment was done by authors KKB and MKB and in case of disagreement, consensus was reached through discussion and independent re-reading of the study in question. With regard to statistical power, the combination of small sample size and lack of assessment of statistical power implied a classification of low statistical conclusion validity. In neuroimaging studies, it is difficult to determine a general “too small” sample size, but in the absence of power analyses, we chose a cutoff of *n* < 20 within the HC group to classify sample size as small.

## Results

### Structural Neuroimaging

**Figure 1:**
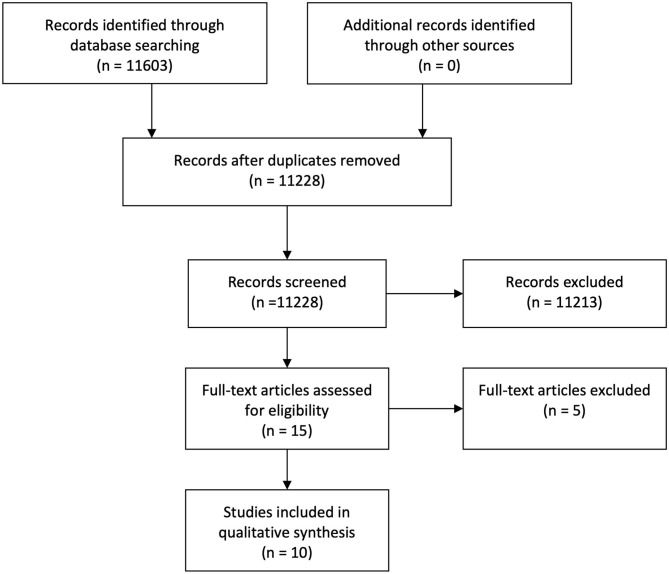
Flowchart Structural Search.

Following the initial exploratory search, a systematic search for structural neuroimaging studies yielded a total of 11,228 hits from the different databases, after removing duplicates. After scanning titles and abstracts, 11,213 citations were excluded. Finally, the full texts were read in order to identify measures and methodological details, further excluding five articles. Thus, 10 articles were deemed eligible for inclusion, based on the aforementioned criteria. No additional articles were found after doing citation searches and reference list reviews. See Figure 1.

### Functional Neuroimaging

**Figure 2:**
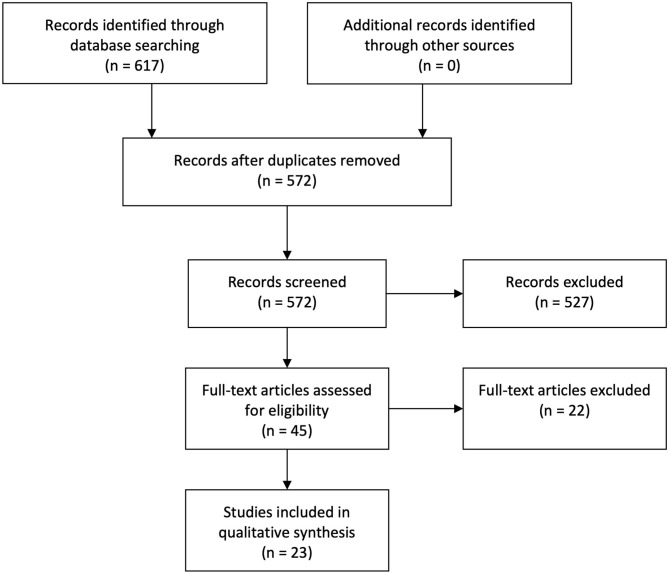
Flowchart functional search.

A second systematic search pursuing functional neuroimaging studies yielded 572 articles, after removing duplicates. A total of 23 articles qualified for inclusion following the same procedures of selection. No additional articles were found after performing citation searches and reference list reviews. See Figure 2.

See Tables 2, 3 for an overview of the included articles.

**Table 2:** Overview of articles concerning the effect of HC on brain structure.

**Reference**	**Sample**	**Type of study and methods**	**Findings**
De Bondt et al. (2013b)	*N* = 30, 15 HC using monophasic oral HC, 15 NC, age span 18–29 years, mean age 21.7, SD 0.5.	Observational combined cross-sectional and RMWC DTI MRI-study. Analyses were ROI based.	Increased diffusivity in fornix in HC group.
De Bondt et al. (2013a)	*N* = 30, 15 HC monophasic oral HC, 15 NC, age span 18–29 years, mean age 21.7, SD 0.5.	Observational RMWC volumetric MRI-study. Whole brain analyses with uncorrected *p*-values were used (*p* < 0.001).	Negatively correlated anterior cingulate gray matter volume with estradiol levels in NC women, not found in HC users.
De Bondt et al. (2015a)	*N* = 75, 37 HC using monophasic oral HC, 38 NC, no information about age (“young women”).	Observational RMWC MRI spectroscopy study.	Increased prefrontal GABA in the NC group at ovulation, but no effects of cycle in HC group. No significant correlations with endogenous hormones or premenstrual symptoms.
De Bondt et al. (2016)	*N* = 75, 38 NC (age-span 18–34 years), 27 androgenic HC (age-span 19–28 years) and 10 anti-androgenic HC (age-span 20–27 years).	Observational RMWC volumetric MRI study. Whole brain analyses with uncorrected *p*-values were used (*p* < 0.001).	In NC women, insula was larger around ovulation than in the luteal phase. Trend effects of HC but did not survive corrections for FWE.
Frokjaer et al. (2009)	*N* = 132, 14 HC (using 20 or 30 mg of ethinylestradiol combined with a progestogen containing 0.10–0.75 mg gestodene, 3 mg drospirenone, or 0.15 mg desogestrel), 15 NC, 87 male controls, mean age 40.6 ± 18.7, age span 18–79 years in full sample, 18–44 in HC group	Observational PET study investigating 5-HT2A receptor binding using [^18^F] altanserin.	No significant effect HC use on cortical 5-HT2A receptor binding.
Lisofsky et al. (2016)	Women planning to start using HC recruited through flyers at gynecologists' offices. *N* = 56, 28 HC (mean age 21.25 years, age span 16–33 years), 28 NC (mean age 21.5 years, age span 16–28 years).	Quasi-experimental pre-post (before and after starting use of HC) volumetric MRI and fMRI-study (no randomization), matched NC controls. Whole brain analyses corrected for FWE were used.	Reduced gray matter volume in left amygdala/anterior parahippocampal gyrus in HC compared to NC group.
Petersen et al. (2015)	*N* = 90, 44 HC using monophasic HC, 46 NC, age-span 18–40 years.	Observational volumetric MRI study. Data analyses entirely ROI based.	Cortical thinning in posterior cingulate and lateral orbitofrontal cortices in women using oral HC compared to NC women.
Pletzer et al. (2010)	*N* = 42, 14 HC (mean age 23.22, SD 3.51), 14 male (mean age 25.30, SD 4.46) 14 NC (mean age 25.88, SD 5.00).	Observational volumetric MRI study. Whole-brain analyses with both uncorrected and FWE corrected *p*-values.	Larger prefrontal cortices, pre- and postcentral gyri, parahippocampal and fusiform gyri in women using HC, compared to NC women.
Pletzer et al. (2015)	*N* = 60, 18 androgenic HC (mean age 24.78, SD 2.94), 22 anti-androgenic HC (mean age 21.95, SD 2.85), 20 NC (mean age 26.60, SD 6.24). 6 controls had never used HC.	Observational volumetric MRI study. Analyses done both with whole brain approach as well as ROI. No significant findings using the whole brain analyses.	Larger gray matter volumes in cerebellum and bilaterally in parahippocampal and fusiform gyri in users of anti-androgenic progestins, middle and superior frontal gyri smaller bilaterally in users of androgenic progestins, both compared to NC women. In NC women who previously used HC, duration of HC use was correlated with hippocampi volumes.
Pletzer (2019)	*N* = 238, 60 HC (mean age 21.42, SD 2.46), 89 NC (mean age 24.02, SD 3.94) and 89 men (mean age: 24.18, SD 4.44 years). Sample overlaps with previous publications.	Observational volumetric MRI study. Analyses done both with FEW corrected whole brain approaches as well as ROI. Associations with self-rated femininity and masculinity investigated.	Whole brain analysis controlling for age and total intracranial volume showed that OC users had significantly smaller regional gray matter than NC women in the right parahippocampal/fusiform gyrus. OC users showed smaller gray matter volumes in the left hippocampus than NC women in the ROI analyses.

*RMWC, Repeated measures within one cycle; HC, hormonal contraceptives; NC, Naturally cycling; EE, ethinyl estradiol; MRI, magnetic resonance imaging; fMRI, functional magnetic resonance imaging; BOLD, blood-oxygen-level dependent; FFA, fusiform face area; SD, standard deviation; MRI, Magnetic resonance imaging; DTI, Diffusion tensor imaging; PET, Positron emission tomography; ROI, Region of interest; FEW, Family-wise error; 5-HT2A, 5-hydroxytryptamine2A; GABA, gamma aminobutyric acid*.

*Information about recruitment is only described if described in the article*.

**Table 3:** Overview of articles concerning the effect of HC on brain function.

**Reference**	**Sample**	**Type of study and methods**	**Findings**
Abler et al. (2013)	University students. *N* = 24, 12 oral HC subjects (4 using 0.03 mg EE with 0.13 mg levonorgestrel or 0.15 mg desogestrel and 8 using 0.03 mg EE combined with 2 mg chlormadinone, 3 mg drospirenone or 2 mg dienogest) and 12 NC subjects. Mean age for total sample: 24.0, age span: 20–29.	Observational RMWC fMRI-study using erotic stimuli. Whole brain analyses with uncorrected *p*-values (*p* < 0.001).	No significant difference in HC vs. NC group upon viewing of explicit erotic stimuli, activation in precentral gyrus increased in NC group during the follicular phase (positively correlated with estrogen levels) upon expectation of erotic stimuli.
Arnoni-Bauer et al. (2017)	*N* = 29, 11 oral HC subjects (Mean age: 26, SD 2) (All using 0.02 mg EE and 0.15 mg desogestrel) and 18 NC subjects (Mean age: 25, SD: 3), age span both groups 18–35.	Observational RMWC fMRI-study using food stimuli. fMRI group comparisons entirely ROI-based.	The HC group had similar fMRI activations in in all activated brain areas as the NC group in the luteal phase but differed from the NC group in the follicular phase.
Basu et al. (2016)	Recruitment through newspaper ads and from a family planning clinic. *N* = 8, subjects were not using HC for the 3 months prior to inclusion. One intramuscular dose of depot medroxyprogesterone acetate was administered to all subjects, age span 18–25.	Quasi-experimental pre-post fMRI study, subjects serving as own controls. fMRI measured before receiving an intramuscular dose of depot medroxyprogesterone acetate and 8 weeks after. Whole brain analyses corrected for FWE.	Increased BOLD response in frontal pole, superior frontal gyrus, orbitofrontal cortex, postcentral gyrus, cingulate cortex, paracingulate gyrus, postcentral gyrus, middle frontal gyrus, superior parietal lobule and lingual gyrus 8 weeks after injection of DMPA vs. baseline.
Bonenberger et al. (2013)	University students. *N* = 24, 12 oral HC subjects (4 using 0.03 mg EE with 0.13 mg levonorgestrel or 0.15 mg desogestrel and 8 using 0.03 mg ethinylestradiol combined with 2 mg chlormadinone, 3 mg drospirenone or 2 mg dienogest) and 12 NC subjects, mean age 24.0, age span 20–29 (same sample as Abler 2013).	Observational RMWC fMRI-study using a monetary incentive task. Whole brain analyses with uncorrected *p*-values (*p* < 0.001) and ROI-analyses.	In ROI based analyses, increased activation in the left anterior insula and inferior lateral prefrontal cortex upon expectation of monetary reward was seen in the HC group as compared to NC group in the follicular phase. No effects of HC in whole brain analyses.
Chung et al. (2016)	*N* = 46, 15 oral HC female subjects, [using estrogen and progestin (or estrogen and an antiandrogen progestin)] 15 NC female subjects and 16 male subjects, mean age 25.46, SD 3.2, age span 19–34.	Observational RMWC MRI-study with regard to effects of HC, but a placebo controlled experimental condition regarding the effects of androstadienone on psychosocial stress responses based on the Montreal Imaging Stress Task. Whole brain FWE-corrected analyses and dorsolateral prefrontal cortex ROI analysis.	No effects of HC. Increased activation of right premotor and supplementary motor areas as well as left somatosensory association cortex during stress in placebo condition, no significant differences during treatment with androstadienone.
De Bondt et al. (2015b)	*N* = 37, 19 oral HC female subjects (using 2nd or 3rd monophasic HC) 18 NC (38 NC and 27 HC before exclusion criteria). No information about age.	Observational RMWC resting state fMRI-study with data extraction using principal component and ICA.	No default mode network differences between any group in any phase were found. There was a positive correlation between functional connectivity in the posterior part of the default mode network and psychological premenstrual-like symptoms seen in inactive pill phase in HC group, not found in NC group.
Gingnell et al. (2013)	Women with previously reported negative affect with oral HC use, recruited through newspaper advertisements. *N* = 30, 15 subjects 30 mcg EE and 0.15 mg levonorgestrel, and 15 placebo, mean age 25.5 +−5 in HC group, 24.5 +−3.3 in placebo group, age span 18–45.	Double-blinded, randomized parallel group clinical trial fMRI-study with pre and post treatment measurement. fMRI paradigm using an emotional face matching task. fMRI group comparisons were entirely ROI-based.	HC women more depressed mood after treatment. Reduced emotion-induced activity in left insula, left middle frontal gyrus and bilateral inferior frontal gyri compared with the NC group and reduced emotion-induced activity in bilateral inferior frontal gyri post HC treatment vs. pre-HC treatment. The placebo group only showed reduced BOLD activity in the amygdala in the last scans.
Gingnell et al. (2016)	Same sample as Gingnell et al. (2013).	Double-blinded, randomized parallel group clinical trial fMRI-study with pre and post treatment measurement. fMRI paradigm using a go/no-go inhibition task. Whole brain analyses, uncorrected for FWE (*p* < 0.001).	Reduced BOLD response in right middle frontal gyrus in HC group, vs. placebo group, no change in behavioral go/no-go performance.
Hornung et al. (2019)	University students. *N* = 67, 29 oral HC female subjects, 20 NC female subjects in luteal phase, 28 male subjects. Mean age 24.07 years, SD 3.64.	Observational fMRI-study using an emotional dot-probe attention-modulation paradigm. Whole brain analyses corrected for FWE and ROI based analyses.	No evidence of differences in neural attentional bias processing between HC and NC group, neither behaviorally nor with regard to brain activity.
Hwang et al. (2015)	*N* = 85, 16 oral HC subjects, 32 NC subjects (median split into 16 with high estradiol and 16 with low estradiol levels) and 37 male subjects, mean age 23.3, SD 2.4 in female group and 29.8, SD 8.8 in male group.	Observational fMRI study using a fear-conditioning and extinction paradigm. FWE-corrected ROI analyses only.	Reduced activation in posterior insula, middle cingulate cortex, amygdala and hypothalamus during a fear conditioning paradigm HC vs. high estrogen NC group. The main findings revolved around the high estrogen group driving most effects.
Lisofsky et al. (2016)	Women planning to start using HCs recruited through flyers at gynecologists' offices. *N* = 56 (28 HC, mean age 21.25 years, age span 16–33 years, 28 NC, mean age 21.5 years, age span 16–28 years.	Quasi-experimental pre-post start use of HC MRI and resting state fMRI-study (no randomization), matched NC controls. Whole brain FWE corrected analyses.	Functional connectivity between left amygdala/anterior parahippocampal gyrus and the right dorsolateral prefrontal cortex changed from positive to negative in the HC group, but findings uncertain due to lack of FWE correction.
Mareckova et al. (2014)	Experiment I: University students *N* = 20, 10 oral HC subjects using 30 mcg EE and 150 mcg levonorgestrel/75 mcg gestodene and 10 NC subjects, mean age 22.0 SD 3.26 in HC group and 20.44 SD 2.69 in NC group, age span 18–29. Experiment II: Adolescents recruited for The IMAGEN European multi-site study through school visits. *N* = 110, 55 oral HC female subjects, 55 NC female subjects, no information about type of oral HC, age span 13.5–15.5.	Observational fMRI-study using movies of either ambiguous or angry faces as stimuli. Whole brain FWE corrected analyses and ROI analyses.	Experiment I: Increased BOLD response to faces in right FFA in HC vs. NC group. Experiment II: Findings replicated. In experiment I, also positive correlation between duration of HC use and the BOLD response in left FFA.
Merz et al. (2012)	*N* = 122, 30 oral HC subjects (using 0.02–0.035 mg EE with a gestagen component, 30 NC subjects in luteal phase, 30 NC subjects in follicular phase, 32 male subjects. Age span 18–35, mean age 21.3–24.8.	Observational fMRI-study using a fear learning paradigm with an experimental condition where cortisol was administered to half the sample. Whole brain FWE corrected analyses and ROI analyses.	Cortisol enhanced fear-learning in HC vs. NC women and there was increased BOLD responses in the left hippocampus and parahippocampal gyrus.
Merz et al. (2013)	University students. *N* = 50, 15 oral HC subjects, 15 NC subjects in the luteal phase, 20 male subjects. Age span 18–35, mean age 23.60, SD 2.13 in HC group, 25.27, SD 3.69 in NC group, 24.15, SD 3.08 in male group.	Observational fMRI study using a fear conditioning paradigm. Whole brain FWE corrected analyses and ROI analyses.	Endogenous cortisol levels positively associated with amygdala BOLD contrasts between CS+ and CS- in men and HC-using women, but not in NC women in the luteal phase.
Miedl et al. (2018)	*N* = 53, 23 oral HC subjects (using EE and a progestin) 30 NC subjects, mean age 22.28, SD 3.73, age span 18–34.	Observational fMRI-study using “traumatic” film viewing as stimuli, with neutral films as reference. All analyses ROI based with corrections for FWE.	Increased BOLD activations in insula and dorsal anterior cingulate cortex during traumatic film viewing in HC groups as compared to NC group.
Monciunskaite et al. (2019)	*N* = 70, 33 combined monophasic anti-androgenic HC users and 37 NC women. Total sample age range 19–38, mean age 23.6, SD 4. No information about recruitment.	Observational EEG/event-related potential study using negative emotional images as stimuli.	HC users showed smaller global field power to visual stimuli than NC women in late latencies (>351 milliseconds). HC users showed blunted late positive potentials to unpleasant stimuli.
Petersen et al. (2014)	Participants recruited from University of California and surrounding community. *N* = 91, 46 oral HC subjects (24 active pill phase, 22 inactive pill phase, HC type consisted of several different types of combined oral contraceptives with different amounts of EE and different types and amount of progestins), 45 NC subjects (20 follicular phase, 25 luteal phase), age-span 18–40.	Observational fMRI resting state study, analyzed using ICA whole brain FWE corrected analyses.	Greater connectivity with the aDMN in left angular gyrus in the follicular NC vs. active phase HC group, increased connectivity with the aDMN in the right caudate nucleus in the follicular group compared to inactive phase HC users. Increased connectivity with ECN in the left anterior cingulate cortex and left middle frontal gyrus seen in follicular group vs. active phase HC group.
Petersen and Cahill (2015)	Same recruitment as Petersen et al. (2014), but included *N* = 83, 40 oral HC subjects (20 active phase, 20 inactive pill phase), 43 NC subjects (20 follicular phase, 23 luteal phase).	Observational fMRI study using 72 negatively valenced, arousing images as stimuli vs. 72 neutral images as reference. Narrow ROI of parts of amygdala.	Reduced bilateral amygdala reactivity in response to negative emotional stimuli seen in HC vs. NC group.
Pletzer et al. (2014)	*N* = 46, 14 oral HC subjects using a combined oral contraceptive, no information about type of estrogen, progestin component consisted of levonorgestrel, other type of 2nd generation progestin or drospirenone, mean age 23.22, SD 3.51, 16 NC subjects, mean age 26.57, SD 6.01, 16 male subjects, mean age 25.14, SD 4.35.	Observational fMRI-study, using number comparison, number bisection. Whole brain FWE corrected analyses.	Different BOLD-activation patterns in HC women as compared to NC women during both numerical tasks, but without any behavioral performance differences. Authors propose that HC women's activation patterns resemble that of men.
Rumberg et al. (2010)	*N* = 36, 12 female subjects using combined oral HC, 12 NC female subjects, 12 male subjects, mean age 31, age span 18–45 in male and NC groups, mean age 23, age span 18–37 in HC group.	Observational fMRI study using a verb-generation task, backwards number counting as reference task. The NC group was scanned twice (ovulatory vs. menstrual period), the other groups were scanned once. Whole brain FWE corrected analyses.	Increased activation during verb generation in right superior temporal cortex in the HC vs. NC group in menstrual phase, and in right inferior frontal cortex in the HC vs. NC group in luteal phase.
Scheele et al. (2016)	*N* = 40, 21 oral HC female subjects, 19 NC female subjects tested in luteal or follicular phase, mean age 24.38, SD 3.26.	Observational fMRI study with regard to HC usage, but randomized, placebo-controlled, double-blind within-group design with regard to effects of oxytocin. Using photographs of partner face, unknown men, familiar women or unfamiliar women. Whole brain FWE corrected analyses.	Oxytocin increased behavioral evaluation of partner attractiveness and BOLD responses in nucleus accumbens and ventral tegmentum in NC group upon viewing partner's face but not in HC group.
Smith et al. (2018)	Recruitment through advertisements. Relevant subset for the *post-hoc* HC analyses: *N* = 28, 17 oral HC subjects, 11 NC subjects.	[18F] fallypride PET-study (targeting dopamine D2/D3 receptor availability) using a dexamphetamine dose as stimuli. Whole brain FWE corrected analyses and ROI analyses.	Plasma estradiol did not correlate with change in dopamine D2/D3 receptor availability and did not differ between HC and NC groups.
Vincent et al. (2013)	Recruitment by advertisement and “word of mouth.” *N* = 24, 12 HC subjects (monophasic combined oral HC) 12 NC subjects, no information about age.	Observational RMWC fMRI-study using noxious thermal stimuli. Whole brain analyses, corrected for FWE and ROI based analyses.	No omnibus effects were found, but in a subgroup of HC users with low testosterone levels, activity in rostral ventromedial medulla was ↓ in HC vs. NC group and this was also associated with lowered pain thresholds.

*RMWC, Repeated measures within one cycle; HC, hormonal contraceptives; NC, naturally cycling; EE, ethinyl estradiol; fMRI, functional magnetic resonance imaging; BOLD, blood-oxygen-level dependent; FFA, fusiform face area, SD: standard deviation, ROI: Region of interest FWE: Family-wise error; DMPA, depomedroxyprogesterone acetate; D2-receptor, Dopamine 2-receptor; D3-receptor, Dopamine 3-receptor; ICA, independent component analysis; aDMN, anterior default mode network; ECN, executive control network; CS, conditioned stimulus*.

*Information about recruitment is only described if described in the article*.

#### Results From the Structural Studies

Most of the included structural studies reported differences between HC users and NC women, as reported in Table 2.

#### Summary of the Structural Studies

All the structural studies tested differences in various brain structures in users of different types of HC as compared to present non-users. The studies were mostly cross-sectional and observational in nature, with the exception of one study (Lisofsky et al., 2016) which was a quasi-experimental pre-post study where a self-selected group of women starting HC use was compared with non-users. However, even in this study, previous use was unaccounted for. Hence no study investigated HC naïve women. The *sample size* ranged from 14 to 60 in the HC groups and from 14 to 89 in the control groups. The *age range* was 18–40 years in both HC and control groups except for the study by Frokjaer et al. (2009) who reported an age range of 18–45 years in the HC group and 18–79 years in a female and male control group. De Bondt et al. (2015a) studied “young women” but did not specify age span. A variety of neuroimaging techniques were employed, including DTI-MRI, volumetric MRI, spectroscopy MRI and PET.

#### HC in Studies on Sex Differences

Several of the studies concerning brain structure were not primarily focused on HC effects on the brain *per se*. Rather, they included HC users in order to investigate whether HC use is an important confounder or moderator in studies on sex differences in the brain. Thus, the aims, methodologies and hypotheses were heterogeneous with regard to HC effects. Four studies (Frokjaer et al., 2009; Pletzer et al., 2010; De Bondt et al., 2016; Pletzer, 2019) explicitly argued that earlier neuroimaging studies on sex differences in the brain did not account for potential confounding effects of HC use in women. These studies assessed brain morphology as related to differential vulnerability to mood and anxiety disorders in men and women. For instance Pletzer et al. (2010), found that NC women had larger prefrontal brain volumes than both men and HC women, and that men had larger hippocampal and amygdalae volumes than women. In a more recent publication, Pletzer pooled and analyzed data from previous publications and noted smaller gray matter volumes in hippocampal and parahippocampal areas in HC users as compared to NC women (Pletzer, 2019). De Bondt et al. (2016) noted that gray matter volumes and PMS symptoms correlated differently in NC and HC groups, whereas Frokjaer et al. (2009) used cortical serotonergic receptor binding as a measure of potential for affective disturbances but discovered no effects of neither sex nor HC use.

Furthermore, as related to whether HC masculinize or feminize brain structure, Pletzer et al. investigated HC effects on the brain depending on the androgenicity of the progestin component of the HC (Pletzer et al., 2015). They found that anti-androgenic progestins promoted larger gray matter volumes in temporal areas such as the fusiform face area and the parahippocampal place area and further related these changes to improved performance in a face recognition task, when comparing with NC women. They also found that users of androgenic progestins had smaller frontal areas compared to NC women.

#### Brain Structures Involved in Cognition and Emotion

A couple of studies specifically focused on HC effects on brain structures known to participate in the processing of emotion and/or cognition. Lisofsky et al. (2016), in a pre-post quasi-experiment with a control group, found decreased gray matter volumes in the amygdala after 3 months of contraceptive intake in women starting HC use after a period on not using HC. They noted that this structural alteration was related to positive affect, whereas no changes in cognitive performance were detected. One study (Petersen et al., 2015) investigated areas involved in the salience network and found cortical thinning in such areas. They were not able, however, to determine whether these changes were causally or merely indirectly related to the use of HC.

#### The Effect of Menstrual Cycle and HC on Brain Structure

One research group has published a series of articles where HC effects were contextualized regarding natural hormonal variation in the menstrual cycle. All these articles had Timo DeBondt as first author. The articles were based on overlapping samples and all assessed the effects of HC as compared to hormonal effects in the menstrual cycle on brain structure (De Bondt et al., 2013a,b, 2015a). Using diffusion tensor imaging, they found a significant increase in mean diffusivity in the fornix in an HC group as compared to a group of NC women (De Bondt et al., 2013b). In the same sample, they also reported that gray matter volume in anterior cingulate cortex (ACC) was negatively associated with estradiol levels in the NC women, whereas this finding could not be replicated in the HC group (De Bondt et al., 2013a). De Bondt et al. (2015a) also examined gamma aminobutyric acid (GABA) concentrations, seeking to find possible correlations between GABA concentration in the PFC, menstrual cycle phase, HC use and premenstrual syndrome (PMS) symptoms. They did find increased prefrontal GABA in the NC group at ovulation, whereas no changes were seen during the cycle in the HC group. No significant correlations with endogenous hormones or PMS symptoms were detected.

#### Adolescent HC Users

None of the structural studies directly investigated effects of HC use on the adolescent brain. Most samples included teenagers from the age of 18, but results were not separated according to age, and as such intermingled with effects on adult brains. This makes it impossible to assess differential or graded effects on younger brains.

#### Results From the Functional Studies

Functional measures were reported in 21 different articles as summarized in Table 3.

#### Summary of Functional Studies

Functional studies were mainly conducted using task based and/or resting state fMRI. In addition, one group used PET and one group EEG. The research groups evaluated cognitive tasks, emotion processing, fear learning, reward and motivation as well as pain inhibition and resting state networks, related to intake of various types of hormonal contraceptives. Only two studies were randomized controlled trials (RCTs) (Gingnell et al., 2013, 2016), whereas the rest were observational, quasi-experimental or observational with repeated measures within one menstrual cycle. *Sample size* range was 8–55 in both HC groups and female control groups. *Age span* was 16–45 years, except in three studies (Vincent et al., 2013; De Bondt et al., 2015b; Smith et al., 2018) which provided no information about age, and four studies where only mean age was provided (Pletzer et al., 2014; Hwang et al., 2015; Scheele et al., 2016; Smith et al., 2018; Hornung et al., 2019). One study (Mareckova et al., 2014) additionally assessed an adolescent sample aged 13.5–15.5 years with 55 participants in both the HC and the NC control group. The functional studies were also heterogenous with regard to aims and approaches as well as design and methodology.

#### Emotion Processing, Fear, Anxiety, and Stress

In line with the scope of some of the structural studies, several of the functional studies investigated brain functions involved in affective processing.

Gingnell et al. (2013) conducted an fMRI RCT with a sample of women with a previous history of HC-induced adverse mood. The subjects were assessed at baseline and once during the last week of the 21 day HC/placebo treatment period. An emotional facial expression matching task was administered. Hemodynamic BOLD (Blood-oxygen-level-dependent) responses to angry or fearful expressions differed between groups and within the HC group when comparing pre-treatment and treatment scans. During the last week of the treatment cycle, the HC group showed decreased reactivity in the bilateral frontal gyri, both compared to the placebo group and to the pre-treatment scans. They also showed decreased reactivity in the left middle frontal gyrus and left insula compared to the placebo women. The changes in brain reactivity were accompanied by more depressed mood, mood swings and fatigue, compared both to the control group and to pre-treatment. The placebo group also showed decreased amygdala reactivity in the last set of scans, whereas this change was not found in the HC group.

Altered amygdala reactivity was also found by Petersen and Cahill (Petersen and Cahill, 2015) who used fMRI to compare reactions related to arousing, negatively valenced images in HC and NC women. They found that HC women had significantly lower amygdala reactivity upon viewing emotionally arousing images.

Investigating the interaction effects of sex hormones and cortisol, Merz et al. (2012) found fMRI activation differences in amygdala, hippocampus and the parahippocampal gyri as a function of interaction of HC use and cortisol administration on implicit emotional learning using a fear learning paradigm. Administration of cortisol reduced amygdala activation in all groups but dampened neural activation in the left hippocampus and in the left anterior parahippocampal gyrus only in NC women. In HC women, hippocampal and parahippocampal activation was enhanced with increased levels of cortisol. In a later study (Merz et al., 2013) Merz et al. evaluated the interaction between endogenous cortisol and the neural correlates of fear expression. There was an interaction between cortisol and HC use, as cortisol levels correlated with BOLD contrasts in the amygdala between conditioned fear stimuli only in HC users.

Fear conditioning was also applied by Hwang et al. (2015), studying fMRI fear responses as well as extinction learning and recall, as related to HC and sex hormone status. HC women had lower activation in the posterior insular cortex, middle cingulate cortex, hypothalamus and amygdala compared to NC women with high levels of estrogen during fear conditioning.

An fMRI “traumatic” film viewing paradigm was utilized by Miedl et al. (2018) to assess the effects of endogenous estradiol and synthetic sex hormones on the neural processing of trauma exposure using films depicting severe interpersonal violence vs. neutral films in NC and HC-using women. The HC group showed increased insula and dorsal ACC activity relative to NC women upon viewing traumatic films.

Two different fMRI studies investigated effects of the pheromone-like steroid androstadienone. Hornung et al. (2019) evaluated differences in attention bias in HC vs. NC women when presented with fearful, angry and happy faces in a “dot probe” task and whether androstadienone affects attention bias. There were no behavioral attentional bias differences, no BOLD response differences and no effects of androstadienone. Similarly, Chung et al. (2016) explored the influence of androstadienone during psychosocial stress in HC, NC and in men using the Montreal Imaging Stress Task. The NC women showed increased activation of the left somatosensory association cortex as well as right pre-motor and supplementary motor areas under the placebo treatment when faced with stress, as compared to HC women. Under treatment with androstadienone, no significant differences were observed between the female groups.

The only included event-related potential (ERP) study was published by Monciunskaite et al. (2019) and employed emotional visual stimuli when comparing women using anti-androgenic HC with NC women. The main finding was that the HC group showed blunted late ERP amplitudes to negative emotional stimuli when compared to NC women.

#### Reward and Motivation

fMRI effects of HC on erotic stimulation and monetary reward was investigated by Abler et al. (2013) and Bonenberger et al. (2013), respectively. Abler et al. presented erotic videos and pictures to HC users and NC women. The MRI scans revealed no between- or within group differences upon viewing these. However, compared to HC users, the NC women in their follicular phase showed increased activation in the bilateral anterior insula, dorsomedial PFC and left inferior parietal lobe, as well as in the bilateral inferior precentral gyrus upon expectation of erotic stimuli. In their luteal phase they had higher activation in the anterior and posterior middle cingulate cortex. Bonenberger et al. examined how the use of HC might alter neural reward processing in a monetary incentive task. In whole-brain analyses, NC and HC women did not differ upon expectation of a monetary reward. An ROI analysis did, however, show enhanced activity in the left anterior insula and inferior lateral PFC in HC users, relative to NC women in their follicular phase.

The interaction of oxytocin and HC regarding perceived partner attractiveness in relation to HC use was studied by Scheele et al. (2016). Subjects were randomized to receive either oxytocin or placebo prior to participating in a passive face-viewing fMRI paradigm. NC and HC pair-bonded women were shown photographs of their romantic partner, matched unknown men, a familiar woman, and a matched unfamiliar woman. Administration of oxytocin was found to enhance ratings of attractiveness of romantic partners compared to unknown men in the NC women, but not in the HC women. NC women showed increased activity in the nucleus accumbens and ventral tegmental area upon viewing their partners, relative to the HC women. The interpretation was that HC can disrupt romantic partner attachment.

HC modulation of fMRI activation upon seeing different food cues was investigated by Arnoni-Bauer et al. (2017) who hypothesized that there would be an association between sex hormones and eating behaviors. Participants were shown images of high calorie foods as well as non-edible items. fMRI activation in the HC group was similar to that of the luteal phase in the NC women. Food related brain activation was assessed also by Basu et al. (2016) who tested the effects of depot medroxyprogesterone acetate (DMPA) on food motivation using a quasi-experimental pre-post design with subjects acting as their own controls. Eight women were investigated with MRI while looking at images of high-calorie and low-calorie foods, as well as neutral, non-food objects. Eight weeks after the DMPA injection increased activation was observed in frontal and postcentral areas upon viewing food, when comparing to baseline. The high-calorie images induced highest activation in cingulate and frontal areas, when comparing to baseline.

A final study of motivational effects of HC was conducted by Smith et al. (2018) who performed a PET study to assess sex differences in dopamine release in inferior frontal areas as well as the dorsal and ventral striatum. They administered D-amphetamine to NC and HC women, as well as to men, to elucidate possible sexually dimorphic neural and hormonal contributions to addiction. They measured changes in dopamine D2 and D3 receptors in the participants, but found no significant effects of HC.

#### Perception of Pain

Vincent et al. (2013) delivered noxious thermal stimuli to HC and NC subjects while in an MRI scanner, aiming to establish whether there was a reduction in the descending pain inhibitory system in the HC group. Serum sex hormone levels were assessed, and participants were asked to rate the intensity of pain for each stimulus delivered. The researchers found that a subgroup of HC women who had decreased testosterone levels required significantly lower temperatures to feel pain, relative to the NC control group. Imaging data showed significantly reduced activity in the rostral ventromedial medulla in response to the noxious stimuli in the low testosterone women, suggesting that failure to engage pain inhibition at this level might be involved in the increased sensitivity to pain in this group. NC women showed higher amygdala activation when compared to high testosterone HC women, but this was not seen when comparing with the low testosterone HC women.

#### Cognitive Tasks

Gingnell et al. (2016) published an fMRI RCT on the effects of HC on brain reactivity during response inhibition, where participants were asked to complete a go/no-go inhibition task. All participants were scanned at baseline and again during the last week of a 21-day treatment cycle. Only the women in the HC group improved performance significantly. HC women showed decreased reactivity in the right orbitofrontal cortex during correct response inhibition. Based on these findings the authors suggest that the use of HC does not necessarily have a negative impact on cognitive control and that, if anything, it might lead to a slight improvement.

Pletzer et al. (2014) assessed fMRI activations during two different numerical tasks which in previous studies had shown systematic sex differences in behavioral performance. HC users were compared to NC women in the follicular and luteal phases of their menstrual cycles, as well as to a group of men. They tested the assumption that brain effects of the synthetic form of progesterone in HC could be induced either by androgenic influences of these progestins (HC group should resemble men), by progestogenic influences (HC group should resemble the luteal group) or through an attenuation of endogenous steroids (HC groups should resemble the follicular group). The HC women resembled the follicular women the most regarding behavioral performance, but their BOLD response resembled that of the men in both cognitive tasks. The main conclusion drawn by the authors was that brain activation patterns in the HC users resembled that of men, but that no behavioral resemblance could be established.

Also employing cognitive tasks in which sex differences have previously been shown, Rumberg et al. (2010) employed fMRi scanning during a verb generation task which consisted of thinking about verbs corresponding to nouns being presented. They found increased activation in the right superior temporal lobe in HC women compared with NC women in their menstrual phase, and in the right inferior frontal cortex comparing with NC women in their mid-cycle phase.

Social cognition was evaluated by Mareckova et al. (2014) in a study on the influence of hormones on face perception. They recruited women using HC as well as NC women and performed fMRI scans while the women were shown ambiguous and angry faces. Both groups underwent fMRI scanning twice, once during the mid-cycle phase and once in the menstrual phase in both groups. Scans revealed stronger BOLD activation in the right fusiform face area in response to both ambiguous and angry faces in the HC groups as compared to the NC group.

#### Resting State and Functional Connectivity

Two of the research groups employed resting state fMRI to study the brain in the absence of tasks. Petersen et al. (2014) measured salivary hormone levels and compared brain activity in the anterior default mode network (DMN) and executive control network (ECN) in early follicular NC women, luteal NC women, HC users in active and inactive pill phases. They found that both endogenous hormone fluctuations and administration of synthetic sex hormones were associated with changes in these networks. De Bondt et al. (2015b) assessed hormone levels as well as symptoms of PMS in NC and HC women in addition to conducting fMRI analyses, but found no significant alterations in the DMN or ECN as a result of neither menstrual cycle phase nor the use of HC. They did, however, observe a positive correlation between PMS-like symptoms in women using HC and functional connectivity in the posterior part of the DMN.

#### Adolescent HC Users

Only one functional study (Mareckova et al., 2014) investigated HC effects on a purely adolescent sample. This sample included teenagers from the age of 13.5–15.5 years. In this study, ROI findings from experiments done on adult participants (Mareckova et al., 2014) were replicated. The teenagers using HC showed increased activity in the left fusiform face area of the temporal lobe upon viewing video clips of faces with ambiguous facial expressions.

## Discussion

In summary, most of the identified neuroimaging studies found effects of HC usage on the female brain, mainly in areas involved in emotional and cognitive processing. However, methodological challenges in almost all the included studies limit our ability to accurately interpret their results and render our main hypotheses to some extent unresolved. The studies by Gingnell et al. (2013, 2016) were the only RCTs concerning the effects of HC. The sample consisted of women with previously reported HC-induced adverse mood, and the articles demonstrated that in women with adverse mood effects, HC may influence negative emotional reactivity and neural networks involved in cognitive inhibition.

Most of the other studies also found effects of HC use on brain structure or function, but these studies had major methodological problems with regard to internal validity or statistical conclusion validity resulting from using familywise uncorrected analyses of MRI-images or small sample sizes. Thus, although we discuss the possible implications of the findings, the reader should keep in mind that these studies are potentially biased. An overview of bias can be found in Supplementary Table 1 and methodological limitations are described in detail in a concluding section. Further, there was only one study with a sample of women in early adolescence, and this was a self-selected convenience sample and hence it may be biased. Thus, our hypothesis regarding effects in adolescence remains unresolved.

### Implications of Structural and Functional Alterations

Most of the included studies indicate that several brain alterations are associated with the use of HC substances. We will discuss the most robust and convergent findings.

Several studies showed effects in areas of the brain known to be implicated in affective processing. Brain mechanisms involving affective changes caused by using of HC are crucial, due to their direct implications for mental health. This point is made convincingly by the register studies by Skovlund et al. showing that HC usage increases depression and suicide risk and that the effects are larger for the youngest women (Skovlund et al., 2016, 2018). According to Gingnell et al. (2013) the use of a combined HC has the potential to negatively affect mood and to induce changes in brain reactivity in structures involved in the processing of fear and other forms of negative affect. In the present review, their studies (Gingnell et al., 2013, 2016) were the strongest in terms of design, and are the only neuroimaging RCTs ever to be performed on functional brain effects of HC. The studies' risk of bias were small, but the researchers only included women with previously reported negative affect in response to the use of HC. Consequently, their sample is not representative for the general female population and external validity is hence limited. However, the study does contribute explanatory findings that are valid for women who experience adverse mood as a side effect of HC use. The women randomized to receive HC showed depressed mood after 1 month of use. This was linked to lower activity in frontal and insular brain areas upon viewing images of angry and fearful facial expressions, as compared to women randomized to receive placebo drugs. In the latter group, less amygdala reactivity was seen in response to images of emotional facial expressions upon a second exposure to these stimuli, whereas a difference upon re-exposure was not seen in women randomized to receive HC drugs. The researchers hypothesized that this might be indicative of decreased amygdala habituation in HC women, and as such attributed the deteriorated mood to an increased vigilance to emotional stimuli.

Further, several other studies in this review, shown in Tables 2, 3, indicate that HC use may affect structures in fear detecting and fear learning circuits in the brain, such as the amygdala. Amygdala functioning is strongly related to fear and learning of fear responses. This is clinically relevant, as fear learning is involved in phobias and other anxiety disorders (Phelps and LeDoux, 2005; Adhikari et al., 2015; Hu et al., 2017). However, the findings are inconsistent, and the studies are heterogenous and confounded by lack of control regarding the androgenic and anti-androgenic effects of the progestins involved. Thus, a balanced interpretation would be that HC use likely affects fear circuits, but that the underlying mechanisms of such effects are not yet understood.

Several studies focused on cognition. The inferior and middle frontal gyri, in particular on the right side of the brain, are associated with inhibition and attentional control (Booth et al., 2005; Aron et al., 2014). In a 2016 RCT, Gingnell et al. (2016) found decreased activity in the right middle frontal gyrus in HC women during a repeated go/no-go inhibition task, both comparing to the pre-treatment cycle and to the NC women. No difference in performance was detected at baseline, but the behavioral performance of the HC women improved more than that of the NC women in the retest session. The authors speculated that this might mean reduced effort in maintaining inhibitory control in the HC women leading to an enhanced inhibitory control in women taking these drugs. Thus, the reduced BOLD activations may be interpreted as increased efficiency and not as an expression of behavioral disinhibition.

Many of the included studies showed effects on the parahippocampal gyrus, both structurally (Pletzer et al., 2010, 2015; Lisofsky et al., 2016) and functionally (Merz et al., 2012; Lisofsky et al., 2016). The parahippocampal gyrus is highly interesting in the context of sex hormones, as it is involved in encoding spatial layout of three-dimensional “scenes” (Furuya et al., 2014). Spatial cognitive ability is one of the cognitive functions where the largest sex differences have been shown (Voyer et al., 1995). However, none of the included studies focused on visuospatial cognition, where functional effects of the identified structural findings would be expected. The structural findings are inconsistent, as Lisofsky et al. (2016) found decreased parahippocampal volume in HC users, whereas Pletzer et al. (2010) found increased volume. Pletzer et al. suggest that an explanation may be that some progestins in HC are androgenic while others are anti-androgenic. They found larger gray matter volumes in the parahippocampal gyri in users of anti-androgenic progestins, but not in users of androgenic progestins, both compared to NC women. The Lisofsky article did not report the specific type of progestin, leaving this inconsistency unresolved.

Facial perception is a process considered to be important for social cognition which is a cognitive function where sex-differences have been found. The fusiform face area plays a role in facial recognition (Axelrod and Yovel, 2015) and effects in this area was reported in the structural studies by Pletzer et al. (2010, 2015) as well as the functional Marečková studies (Mareckova et al., 2014) conducted with adult and adolescent samples. These studies found increased BOLD response in the fusiform face area upon viewing ambiguous and angry faces. The Marečková findings also provide a link between duration of HC use and extent of impact on the brain, as the activity in this area was increased as a function of length of use. The authors suggest a long-term plastic adaptation of the brain related to the use of HC. Thus, HC may influence social cognition, although the functional implications are unresolved.

Several research groups found functional effects of HC use in areas involved in the regulation of reward and motivation. The researchers used food-related, romantic, and sexual as well as monetary stimuli as a means of measuring such effects. The most important areas in the brain regarding reward, involve the dopaminergic mesolimbic structures such as nucleus accumbens in the striatum as well as the ventral tegmental area (VTA) (Arias-Carrion et al., 2010). Oxytocin-releasing neurons terminate on these areas and oxytocin is thought to mediate reward (Peris et al., 2017). Changes in these systems may affect all forms of motivated behaviors, thus having important effects in all areas of life. For instance, the study by Scheele et al. (2016) which assessed perceived partner attractiveness, found that upon viewing the partner's face, treatment with oxytocin increased the behavioral evaluation of partner attractiveness as well as BOLD responses in the nucleus accumbens and the VTA, in the NC group. This was not found in the HC group. The possible implication is that HC may attenuate partner-bonding. This remains speculative but should be explored further due to the seriousness of the potential consequences. The studies on sexual, monetary and food-related rewards (Abler et al., 2013; Bonenberger et al., 2013; Basu et al., 2016) suffer from possible retest effects in only some of the subjects, *post-hoc* finding present only in an ROI based analysis and a small sample, respectively, thus presenting with reduced validity.

### Lack of Pure Adolescent Samples

In addition to hypothesizing about the ability of HC to affect structural and functional aspects of the brain, we expected effects to be larger in adolescent subjects than in adult subjects. However, as we identified only one neuroimaging study ever to be performed on a purely adolescent sample, this hypothesis remains unresolved and the effects of such drugs on developing brains remain undetermined. The studies included many older subjects, making it impossible to disentangle potential differences between effects on the adolescent brain and effects on the adult brain. None of the studies investigated structural changes related to the use of HC in drug-naïve teenagers, but rather included convenience samples with mostly adult subjects. Only one functional study (Mareckova et al., 2014) included a strictly adolescent sample, but there was no direct comparison with older subjects, nor any statistical test of age-covariates.

Given the evidence from the animal literature, as well as clinical registry studies such as that by Skovlund et al. (2016, 2018), which strongly indicate an increased vulnerability of the brain during adolescence, combined with the fact that girls are using these substances from an early adolescent age, we argue that there is a strong need for future studies to be carried out on adolescent use of HC.

### Methodological Limitations in the Included Studies

We applied the validity typology of Donald Campbell and Thomas D. Cook (Cook et al., 1979) which encompasses 4 types of validity threats with regard to our ability to make causal inferences: Internal validity, external validity, statistical conclusion validity and construct validity. While all types are important, low internal validity is paramount as is concerns whether an intervention was the likely cause of an effect. Thus, internal validity mainly encompasses confounders. See Supplementary Table 1 for a summary of the quality evaluation.

With the exception of Gingnell et al. (2013, 2016), none of the studies randomized participants to receive either HC or placebo, and most of the studies were observational with no inclusion of HC-naïve women. Hence, only the Gingnell studies reached high internal validity. The combined structural and functional MRI study by Lisofsky et al. (2016) achieved intermediate internal validity as they employed a pre-post quasi-experiment with control group, because even though the subjects self-selected to use HC, risk of bias was lowered due to the longitudinal design, enabling comparisons of within and between group effects. Yet, this design cannot control for effects of previous use. While this is true also for Gingnell, they explicitly aimed to generalize to a population of previous users. Thus, as stated previously, the Gingnell study cannot be generalized to the population of all women.

The conclusion regarding internal validity is that all studies, except the ones by Gingnell et al. were susceptible to bias and confounding due to selection phenomena and unobserved variables. Convenience sampling without disclosed detail concerning recruitment, as well as lack of randomization and control groups in almost all of the included studies, makes it impossible to ascertain causality.

Furthermore, most studies had poor control regarding type of substance currently or previously used, and no control for age at start of previous use, leading to low external validity. This critique also pertain to the Gingnell RCTs, as it is only possible to generalize to women with previous negative mood effects while using HC.

Most studies had low statistical conclusion validity, with small samples, resulting in low statistical power, making negative findings difficult to interpret, but also to an increased risk of false positive results (Button et al., 2013). Many of the findings were also based on ROI analyses without familywise error (FWE) corrected whole brain analyses. ROI areas can be chosen based on *post-hoc* considerations, and so there should be a strong theoretical and/or empirical basis for choice of ROI areas. Several studies also employed whole brain analyses without correction for FWE. This may have led to type 1 errors.

Thus, while most studies found effects of HC on brain function or structure, confounding cannot be ruled out. While different studies had different methodological problems, the main source of low validity was self-selection in all of these studies, with the exception of the Gingnell studies. Thus, we discuss the effects of self-selection in the next paragraph.

### The Impact of Sampling Bias and Self-Selection

Self-selection is a major internal validity threat in all of the non-randomized studies and is highly problematic in the present context. Choosing or not choosing to use HC may be influenced by various psychological factors that are associated with differences in brain structure and function. Mental and behavioral functions are, to a large extent, determined by brain function which ultimately is determined by brain structure. Thus, in the absence of randomization, self-selection by choosing or not choosing to use contraceptive drugs could be caused by psychological factors that are at least partly determined by brain function or structure. This could lead to serious confounding that could threaten internal validity.

Delayed sexual debut or sexual abstinence are examples of behaviors that may in part be determined by differences in brain function or structure when contrasted with being sexually active. Personality factors such as extraversion are central in this regard. In a large Dutch study, extraversion was found to affect friendships which again affected sexual debut and behavior (van Leeuwen and Mace, 2016). A meta-analysis including altogether 420,595 subjects showed that extraversion was clearly positively associated with sexual activity (Allen and Walter, 2018). Extraversion is further associated with distinct resting state fMRI patterns, such as increased long-range functional connectivity (Pang et al., 2017). Structurally, it is associated with smaller gray matter volumes in the bilateral basal ganglia and increased dopamine receptor density in the striatum (Baik et al., 2012). Also, negative associations with right PFC volumes have been found (Forsman et al., 2012). This exemplifies how closely sexual activity is related to personality, which is further associated with differences both in brain function and structure. It thus illustrates how self-selection may have seriously confounded the included studies.

Another important source of possible bias is discontinued use of HC due to negative side effects. Different women may experience different side effects, and if such effects are not independent from brain function or structure, this will bias the finding. Thus, women who have chosen not to continue using HC will not be included in studies on effects of such drugs, unless the design of the study is a randomized design, and not based on self-selection.

As almost all the included studies were non-randomized case control-studies they might have ignored factors like these, and this might have introduced a strong sampling or selection bias. If the researchers had used only drug-naïve subjects for both controls and HC users, one could eliminate possible confounding effects of earlier use on their brains. By also employing longitudinal designs with drug-naïve subjects and pre-usage measures of brain-behavior relationships, validity could be further increased.

### Contraceptive Content and Routes of Administration

There is a wide variety of HC drugs available, and these might affect the female brain in different ways. The orally administered drugs can be combination pills that commonly consist of ethinylestradiol and a progestin, or progestin-only formulations. They may have different cycle regimens, such as mono-, bi-, tri-, and quadriphasic as well as flexible regimens. Both the estrogen and the progestin contents of these pills have been gradually lowered over the years in an effort to reduce side effects (Christin-Maitre, 2013).

Different types of formulation may also be associated with different side effects. Some progestins are considered to have androgenic properties, while others may have anti-androgenic effects on brain and behavior (Pletzer and Kerschbaum, 2014; Giatti et al., 2016). Progesterone may lead to reduced testosterone action due to affinity for the enzyme 5α-reductase, and this may reduce conversion of testosterone into the more potent dihydrotestosterone (Pletzer and Kerschbaum, 2014). Combined oral contraceptives with a progestin content considered to be anti-androgenic, such as drospirenone and desogestrel, have been postulated to be favorable in terms of mood symptoms in comparison with progestins displaying an androgenic profile (Poromaa and Segebladh, 2012).

Alternative administration routes have also been developed over the years, such as vaginal or transdermal. Long-acting reversible contraception (LARC) such as progestogen-releasing intrauterine devices as well as injectable substances and implantable devices are effective contraceptive options that have become increasingly popular in the past decades (Kavanaugh et al., 2015). Several of the included studies have recruited participants not using the same drug and/or using different routes of administration, and other studies do not provide information about these variables. This introduces the chance of committing type II errors and hence neglecting to uncover effects of the given drugs, since other drugs studied simultaneously, but having a different profile, may have counteracted or canceled out the effects on a group level.

## Conclusions

This review found evidence that the use of HC can alter both structure and function of the brain. Furthermore, it contributed to accentuating the need for future research on HC and the ways in which they may affect the brain. There is a need for systematic research that considers the differences in formulation and administration of the various contraceptive drugs, employing a longitudinal, within-subject design with matched and randomized control groups consisting of HC-naïve subjects.

The impact of structural changes in the brain on functional outcomes such as motivational factors, affective phenomena and cognitive abilities should indeed be further investigated. Given the well-known sex hormone-dependent brain plasticity (Schulz and Sisk, 2016), adolescence may be seen as a window of both increased opportunity and increased vulnerability, where implications of interference with endogenous processes could be far-reaching and affect emotional, relational, educational and vocational aspects of life. As a substantial number of women start using HC at a young age (Martinez et al., 2020), these are issues that need to be scientifically addressed in order to provide female adolescents with individualized and informed contraceptive choices.

## Author Contributions

MB: initial draft. MB and KB: conception/design and acquisition. All authors: analysis, interpretation of data, revision, final approval, and agreement to be accountable for all aspects of the work.

## Conflict of Interest

The authors declare that the research was conducted in the absence of any commercial or financial relationships that could be construed as a potential conflict of interest.

## References

[B1] Abler B. Kumpfmüller D. Grön G. Walter M. Stingl J. Seeringer A. (2013). neural correlates of erotic stimulation under different levels of female sexual hormones. PLoS ONE 8:e0054447. 10.1371/journal.pone.0054447 23418428 PMC3572100

[B2] Adhikari A. Lerner T. N. Finkelstein J. Pak S. Jennings J. H. Davidson T. J.. (2015). Basomedial amygdala mediates top-down control of anxiety and fear. Nature 527, 179–185. 10.1038/nature15698 26536109 PMC4780260

[B3] Allen M. S. Walter E. E. (2018). Linking big five personality traits to sexuality and sexual health: A meta-analytic review. Psychol. Bull. 144, 1081–1110. 10.1037/bul0000157 29878796

[B4] Arias-Carrion O. Stamelou M. Murillo-Rodriguez E. Menendez-Gonzalez M. Poppel E. (2010). Dopaminergic reward system: a short integrative review. Int. Arch. Med. 3:24. 10.1186/1755-7682-3-24 20925949 PMC2958859

[B5] Arnoni-Bauer Y. Bick A. Raz N. Imbar T. Amos S. Agmon O.. (2017). Is it me or my hormones? neuroendocrine activation profiles to visual food stimuli across the menstrual cycle. J. Clin. Endocrinol. Metaboli. 102, 3406–3414. 10.1210/jc.2016-3921 28911135

[B6] Aron A. R. Robbins T. W. Poldrack R. A. (2014). Inhibition and the right inferior frontal cortex: one decade on. Trends Cogn. Sci. 18, 177–185. 10.1016/j.tics.2013.12.003 24440116

[B7] Axelrod V. Yovel G. (2015). Successful decoding of famous faces in the fusiform face area. PLoS ONE 10:e0117126. 10.1371/journal.pone.0117126 25714434 PMC4340964

[B8] Baik S. H. Yoon H. S. Kim S. E. Kim S. H. (2012). Extraversion and striatal dopaminergic receptor availability in young adults: an [18F]fallypride PET study. Neuroreport 23, 251–254. 10.1097/WNR.0b013e3283507533 22257904

[B9] Basu T. Bao P. Lerner A. Anderson L. Page K. Stanczyk F.. (2016). The effect of depo medroxyprogesterone acetate (dmpa) on cerebral food motivation centers: a pilot study using functional magnetic resonance imaging. Contraception 94, 321–327. 10.1016/j.contraception.2016.04.011 27129935

[B10] Beltz A. M. Berenbaum S. A. (2013). Cognitive effects of variations in pubertal timing: is puberty a period of brain organization for human sex-typed cognition? Hormones behav. 63, 823–828. 10.1016/j.yhbeh.2013.04.002 23603479

[B11] Beltz A. M. Hampson E. Berenbaum S. A. (2015). Oral contraceptives and cognition: a role for ethinyl estradiol. Hormone. Behav. 74, 209–217. 10.1016/j.yhbeh.2015.06.012 26122296

[B12] Blakemore S.-J. (2012). Imaging brain development: the adolescent brain. Neuroimage. 61, 397–406. 10.1016/j.neuroimage.2011.11.080 22178817

[B13] Bonenberger M. Groschwitz R. C. Kumpfmueller D. Groen G. Plener P. L. Abler B. (2013). It's all about money: Oral contraception alters neural reward processing. NeuroReport 24, 951–955. 10.1097/WNR.0000000000000024 24136199

[B14] Booth J. R. Burman D. D. Meyer J. R. Lei Z. Trommer B. L. Davenport N. D.. (2005). Larger deficits in brain networks for response inhibition than for visual selective attention in attention deficit hyperactivity disorder (ADHD). J. Child Psychol. Psychiatr. 46, 94–111. 10.1111/j.1469-7610.2004.00337.x 15660647

[B15] Button K. S. Ioannidis J. P. Mokrysz C. Nosek B. A. Flint J. Robinson E. S.. (2013). Power failure: why small sample size undermines the reliability of neuroscience. Nat. Rev.Neurosci. 14, 365–376. 10.1038/nrn3475 23571845

[B16] Cahill L. (2006). Why sex matters for neuroscience. Nat. Rev. Neurosci. 7:477. 10.1038/nrn1909 16688123

[B17] Christin-Maitre S. (2013). History of oral contraceptive drugs and their use worldwide. Best Prac. Res. Clin. Endocrinol. Metabol. 27, 3–12. 10.1016/j.beem.2012.11.004 23384741

[B18] Chung K. C. Springer I. Kogler L. Turetsky B. Freiherr J. Derntl B. (2016). The influence of androstadienone during psychosocial stress is modulated by gender, trait anxiety and subjective stress: An fMRI study. Psychoneuroendocrinology 68:126–139. 10.1016/j.psyneuen.2016.02.026 26970712

[B19] Comasco E. Hahn A. Ganger S. Gingnell M. Bannbers E. Oreland L.. (2014). Emotional fronto-cingulate cortex activation and brain derived neurotrophic factor polymorphism in premenstrual dysphoric disorder. Hum. Brain Mapp. 35, 4450–4458. 10.1002/hbm.22486 24615932 PMC4107029

[B20] Cook T. D. Campbell D. T. McCleary R. McCain L. J. Reichardt C. S. Fankhauser G.. (1979). Quasi-Experimentation : Design Analysis Issues For Field Settings. Boston, MA: Houghton Mifflin Co.

[B21] Daniels K. Jones J. (2013). Contraceptive Methods Women Have Ever Used: United States, 1982-2010: US Department of Health and Human Services, Centers for Disease Control and Prevention. Los Angeles, CA: National Center for Health Statistics.

[B22] De Bondt T. De Belder F. Vanhevel F. Jacquemyn Y. Parizel P. M. (2015a). Prefrontal GABA concentration changes in women-Influence of menstrual cycle phase, hormonal contraceptive use, and correlation with premenstrual symptoms. Brain Res. 1597, 129–138. 10.1016/j.brainres.2014.11.051 25481417

[B23] De Bondt T. Jacquemyn Y. Van Hecke W. Sijbers J. Sunaert S. Parizel P. M. (2013a). Regional gray matter volume differences and sex-hormone correlations as a function of menstrual cycle phase and hormonal contraceptives use. Brain Res. 1530:22–31. 10.1016/j.brainres.2013.07.034 23892107

[B24] De Bondt T. Pullens P. Van Hecke W. Jacquemyn Y. Parizel P. M. (2016). Reproducibility of hormone-driven regional grey matter volume changes in women using SPM8 and SPM12. Brain Struc. Func. 221, 4631–4641. 10.1007/s00429-016-1193-1 26862108

[B25] De Bondt T. Smeets D. Pullens P. Van Hecke W. Jacquemyn Y. Parizel P. M. (2015b). Stability of resting state networks in the female brain during hormonal changes and their relation to premenstrual symptoms. Brain Res. 1624, 275–285. 10.1016/j.brainres.2015.07.045 26253822

[B26] De Bondt T. Van Hecke W. Veraart J. Leemans A. Sijbers J. Sunaert S.. (2013b). Does the use of hormonal contraceptives cause microstructural changes in cerebral white matter? Preliminary results of a DTI and tractography study. Eur. Radiol. 23, 57–64. 10.1007/s00330-012-2572-5 22814829

[B27] Forsman L. J. de Manzano O. Karabanov A. Madison G. Ullen F. (2012). Differences in regional brain volume related to the extraversion-introversion dimension–a voxel based morphometry study. Neurosci. Res. 72, 59–67. 10.1016/j.neures.2011.10.001 22008612

[B28] Frokjaer V. G. Erritzoe D. Madsen J. Paulson O. B. Knudsen G. M. (2009). Gender and the use of hormonal contraception in women are not associated with cerebral cortical 5-HT 2A receptor binding. Neuroscience 163, 640–645. 10.1016/j.neuroscience.2009.06.052 19559762

[B29] Fuhrmann D. Knoll L. J. Blakemore S.-J. (2015). Adolescence as a sensitive period of brain development. Trends Cogn. Sci. 19, 558–566. 10.1016/j.tics.2015.07.008 26419496

[B30] Furuya Y. Matsumoto J. Hori E. Boas C. V. Tran A. H. Shimada Y.. (2014). Place-related neuronal activity in the monkey parahippocampal gyrus and hippocampal formation during virtual navigation. Hippocampus 24, 113–30. 10.1002/hipo.22209 24123569

[B31] Garcia N. Walker R. Zoellner L. (2018). Estrogen, progesterone, and the menstrual cycle: A systematic review of fear learning, intrusive memories, and PTSD. Clin. Psychol. Rev. 66, 80–96. 10.1016/j.cpr.2018.06.005 29945741

[B32] Giatti S. Melcangi R. C. Pesaresi M. (2016). The other side of progestins: effects in the brain. J. Mol. Endocrinol. 57, R109–R126. 10.1530/JME-16-0061 27339142

[B33] Gingnell M. Bannbers E. Engman J. Frick A. Moby L. Wikström J.. (2016). The effect of combined hormonal contraceptives use on brain reactivity during response inhibition. Eur. J. Contr. Reprod. Health Care. 21, 150–157. 10.3109/13625187.2015.1077381 26291330

[B34] Gingnell M. Engman J. Frick A. Moby L. Wikstrom J. Fredrikson M.. (2013). Oral contraceptive use changes brain activity and mood in women with previous negative affect on the pill-A double-blinded, placebo-controlled randomized trial of a levonorgestrel-containing combined oral contraceptive. Psychoneuroendocrinology. 38, 1133–1144. 10.1016/j.psyneuen.2012.11.006 23219471

[B35] Griksiene R. Monciunskaite R. Arnatkeviciute A. Ruksenas O. (2018). Does the use of hormonal contraceptives affect the mental rotation performance? Hormone. Behav. 100, 29–38. 10.1016/j.yhbeh.2018.03.004 29522764

[B36] Herting M. M. Gautam P. Spielberg J. M. Kan E. Dahl R. E. Sowell E. R. (2014). The role of testosterone and estradiol in brain volume changes across adolescence: a longitudinal structural MRI study. Hum. Brain Mapp. 35, 5633–5645. 10.1002/hbm.22575 24977395 PMC4452029

[B37] Hines M. (2006). Prenatal testosterone and gender-related behaviour. Eur. J. Endocrinol. 155(suppl_1), S115–S121. 10.1530/eje.1.02236 17074984

[B38] Hornung J. Noack H. Kogler L. Derntl B. (2019). Exploring the fMRI based neural correlates of the dot probe task and its modulation by sex and body odor. Psychoneuroendocrinology 99, 87–96. 10.1016/j.psyneuen.2018.08.036 30216766

[B39] Hu Y. Moore M. Bertels Z. Phan K. L. Dolcos F. Dolcos S. (2017). Smaller amygdala volume and increased neuroticism predict anxiety symptoms in healthy subjects: a volumetric approach using manual tracing. Neuropsychologia 145:106564. 10.1016/j.neuropsychologia.2017.11.008 29157997

[B40] Hwang M. J. Zsido R. G. Song H. Pace-Schott E. F. Miller K. K. Lebron-Milad K.. (2015). Contribution of estradiol levels and hormonal contraceptives to sex differences within the fear network during fear conditioning and extinction. BMC Psychiatr. 15:9. 10.1186/s12888-015-0673-9 26581193 PMC4652367

[B41] Josso N. (2008). Professor alfred jost: the builder of modern sex differentiation. Sexual Dev. 2, 55–63. 10.1159/000129690 18577872

[B42] Kavanaugh M. L. Jerman J. Finer L. B. (2015). Changes in use of long-acting reversible contraceptive methods among US women, 2009–2012. Obstet. Gynecol. 126:917–927. 10.1097/AOG.0000000000001094 26444110 PMC4946164

[B43] Lisofsky N. Riediger M. Gallinat J. Lindenberger U. Kuhn S. (2016). Hormonal contraceptive use is associated with neural and affective changes in healthy young women. NeuroImage. 134:597–606. 10.1016/j.neuroimage.2016.04.042 27109356

[B44] Mareckova K. Perrin J. S. Khan I. N. Lawrence C. Dickie E. McQuiggan D. A.. (2014). Hormonal contraceptives, menstrual cycle and brain response to faces. Soc. Cogn. Affect. Neurosci. 9, 191–200. 10.1093/scan/nss128 23175677 PMC3907931

[B45] Martinez G. Copen C. E. Abma J. C. (2020). Teenagers in the United States: sexual activity, contraceptive use, and childbearing, 2006-2010 national survey of family growth. Vital Health Stat. 23, 1–35. 22256688

[B46] McCarthy M. M. Nugent B. M. (2015). At the frontier of epigenetics of brain sex differences. Front. Behav. Neurosci. 9:221. 10.3389/fnbeh.2015.00221 26347630 PMC4543874

[B47] Merz C. J. Stark R. Vaitl D. Tabbert K. Wolf O. T. (2013). Stress hormones are associated with the neuronal correlates of instructed fear conditioning. Biol. Psychol. 92, 82–89. 10.1016/j.biopsycho.2012.02.017 22406758

[B48] Merz C. J. Tabbert K. Schweckendiek J. Klucken T. Vaitl D. Stark R.. (2012). Oral contraceptive usage alters the effects of cortisol on implicit fear learning. Hormon Behav. 62, 531–538. 10.1016/j.yhbeh.2012.09.001 22986336

[B49] Miedl S. F. Wegerer M. Kerschbaum H. Blechert J. Wilhelm F. H. (2018). Neural activity during traumatic film viewing is linked to endogenous estradiol and hormonal contraception. Psychoneuroendocrinology 87, 20–26. 10.1016/j.psyneuen.2017.10.006 29032323

[B50] Moher D. Liberati A. Tetzlaff J. Altman D. G. Group P. (2009). Preferred reporting items for systematic reviews and meta-analyses: the PRISMA statement. PLoS Med. 6:e1000097 10.1371/journal.pmed.1000097 19621072 PMC2707599

[B51] Monciunskaite R. Malden L. Lukstaite I. Ruksenas O. Griksiene R. (2019). Do oral contraceptives modulate an ERP response to affective pictures? Biol. Psychol. 148:107767. 10.1016/j.biopsycho.2019.107767 31509765

[B52] Norwegian Prescription Database (2019). The Norwegian Institute of Public Health. Available online at: www.reseptregisteret.no(accessed February 01, 2020).

[B53] Pang Y. Cui Q. Duan X. Chen H. Zeng L. Zhang Z.. (2017). Extraversion modulates functional connectivity hubs of resting-state brain networks. J. Neuropsychol. 11, 347–361. 10.1111/jnp.12090 26566723

[B54] Pascual-Leone A. Amedi A. Fregni F. Merabet L. B. (2005). The plastic human brain cortex. Annu. Rev. Neurosci. 28:377–401. 10.1146/annurev.neuro.27.070203.144216 16022601

[B55] Peper J. Pol H. H. Crone E. Van Honk J. (2011). Sex steroids and brain structure in pubertal boys and girls: a mini-review of neuroimaging studies. Neuroscience 191, 28–37. 10.1016/j.neuroscience.2011.02.014 21335066

[B56] Peris J. MacFadyen K. Smith J. A. de Kloet A. D. Wang L. Krause E. G. (2017). Oxytocin receptors are expressed on dopamine and glutamate neurons in the mouse ventral tegmental area that project to nucleus accumbens and other mesolimbic targets. J. Comp. Neurol. 525, 1094–1108. 10.1002/cne.24116 27615433 PMC6483090

[B57] Petanjek Z. Judaš M. Šimi C. G. Rašin M. R. Uylings H. B. Rakic P.. (2011). Extraordinary neoteny of synaptic spines in the human prefrontal cortex. Proc. Natl. Acad. Sci. U.S.A. 108, 13281–13286. 10.1073/pnas.1105108108 21788513 PMC3156171

[B58] Petersen N. Cahill L. (2015). Amygdala reactivity to negative stimuli is influenced by oral contraceptive use. Soc. Cogn. Affect. Neurosci. 10, 1266–1272. 10.1093/scan/nsv010 25688096 PMC4560944

[B59] Petersen N. Kilpatrick L. A. Goharzad A. Cahill L. (2014). Oral contraceptive pill use and menstrual cycle phase are associated with altered resting state functional connectivity. NeuroImage 90, 24–32. 10.1016/j.neuroimage.2013.12.016 24365676 PMC4113343

[B60] Petersen N. Touroutoglou A. Andreano J. M. Cahill L. (2015). Oral contraceptive pill use is associated with localized decreases in cortical thickness. Hum. Brain Mapp. 36, 2644–2654. 10.1002/hbm.22797 25832993 PMC4478200

[B61] Phelps E. A. LeDoux J. E. (2005). Contributions of the amygdala to emotion processing: from animal models to human behavior. Neuron 48, 175–187. 10.1016/j.neuron.2005.09.025 16242399

[B62] Phoenix C. H. Goy R. W. Gerall A. A. Young W. C. (1959). Organizing action of prenatally administered testosterone propionate on the tissues mediating mating behavior in the female guinea pig. Endocrinology. 65, 369–382. 10.1210/endo-65-3-369 14432658

[B63] Pletzer B. (2019). Sex hormones and gender role relate to gray matter volumes in sexually dimorphic brain areas. Front Neurosci. 13:592. 10.3389/fnins.2019.00592 31275099 PMC6591487

[B64] Pletzer B. Kronbichler M. Aichhorn M. Bergmann J. Ladurner G. Kerschbaum H. H. (2010). Menstrual cycle and hormonal contraceptive use modulate human brain structure. Brain Res. 1348:55–62. 10.1016/j.brainres.2010.06.019 20550945

[B65] Pletzer B. Kronbichler M. Kerschbaum H. (2015). Differential effects of androgenic and anti-androgenic progestins on fusiform and frontal gray matter volume and face recognition performance. Brain Res. 1596, 108–115. 10.1016/j.brainres.2014.11.025 25446458

[B66] Pletzer B. Kronbichler M. Nuerk H.-C. Kerschbaum H. (2014). Hormonal contraceptives masculinize brain activation patterns in the absence of behavioral changes in two numerical tasks. Brain Res. 1543:128–142. 10.1016/j.brainres.2013.11.007 24231554

[B67] Pletzer B. A. Kerschbaum H. H. (2014). 50 years of hormonal contraception-time to find out, what it does to our brain. Front. Neurosci. 8:256. 10.3389/fnins.2014.00256 25191220 PMC4139599

[B68] Poromaa I. S. Segebladh B. (2012). Adverse mood symptoms with oral contraceptives. Acta Obstet. Gynecol. Scand. 91, 420–427. 10.1111/j.1600-0412.2011.01333.x 22136510

[B69] Rumberg B. Baars A. Fiebach J. Ladd M. E. Forsting M. Senf W.. (2010). Cycle and gender-specific cerebral activation during a verb generation task using fMRI: Comparison of women in different cycle phases, under oral contraception, and men. Neurosci. Res. 66, 366–371. 10.1016/j.neures.2009.12.011 20036289

[B70] Scheele D. Plota J. Stoffel-Wagner B. Maier W. Hurlemann R. (2016). Hormonal contraceptives suppress oxytocin-induced brain reward responses to the partner's face. Soc. Cogn. Affect. Neurosci. 11, 767–774. 10.1093/scan/nsv157 26722017 PMC4847696

[B71] Schulz K. M. Sisk C. L. (2016). The organizing actions of adolescent gonadal steroid hormones on brain and behavioral development. Neurosci. Biobehav. Rev. 70,148–158. 10.1016/j.neubiorev.2016.07.036 27497718 PMC5074860

[B72] Sherman L. E. Rudie J. D. Pfeifer J. H. Masten C. L. McNealy K. Dapretto M. (2014). Development of the default mode and central executive networks across early adolescence: a longitudinal study. Dev. Cogn. Neurosci. 10,148–159. 10.1016/j.dcn.2014.08.002 25282602 PMC4854607

[B73] Skovlund C. W. Morch L. S. Kessing L. V. Lange T. Lidegaard O. (2018). Association of hormonal contraception with suicide attempts and suicides. Am. J. Psychiatr. 175, 336–342. 10.1176/appi.ajp.2017.17060616 29145752

[B74] Skovlund C. W. Morch L. S. Kessing L. V. Lidegaard O. (2016). Association of hormonal contraception with depression. JAMA Psychiatr. 73, 1154–1162. 10.1001/jamapsychiatry.2016.2387 27680324

[B75] Smith C. T. Dang L. C. Burgess L. L. Perkins S. F. San Juan M. D. Smith D. K.. (2018). Lack of consistent sex differences in d-amphetamine-induced dopamine release measured with [18f]fallypride pet. Psychopharmacology. 236, 581–590. 10.1007/s00213-018-5083-5 30350220 PMC6401232

[B76] Toffoletto S. Lanzenberger R. Gingnell M. Sundström-Poromaa I. Comasco E. (2014). Emotional and cognitive functional imaging of estrogen and progesterone effects in the female human brain: a systematic review. Psychoneuroendocrinology 50, 28–52. 10.1016/j.psyneuen.2014.07.025 25222701

[B77] van Leeuwen A. J. Mace R. (2016). Life history factors, personality and the social clustering of sexual experience in adolescents. R. Soc. Open Sci. 3:160257. 10.1098/rsos.160257 27853543 PMC5098968

[B78] Vincent K. Warnaby C. Stagg C. J. Moore J. Kennedy S. Tracey I. (2013). Brain imaging reveals that engagement of descending inhibitory pain pathways in healthy women in a low endogenous estradiol state varies with testosterone. Pain. 154, 515–524. 10.1016/j.pain.2012.11.016 23318125

[B79] Voyer D. Voyer S. Bryden M. P. (1995). Magnitude of sex differences in spatial abilities: a meta-analysis and consideration of critical variables. Psychol. Bull. 117:250. 10.1037/0033-2909.117.2.250 7724690

[B80] Wai J. Cacchio M. Putallaz M. Makel M. C. (2010). Sex differences in the right tail of cognitive abilities: A 30 year examination. Intelligence 38, 412–423. 10.1016/j.intell.2010.04.006

