# Supplementary material for: The Effects of Hormonal Contraceptives on the Brain: A Systematic Review of Neuroimaging Studies
Source: Front Psychol. 2020 Oct 27;11:556577. doi: 10.3389/fpsyg.2020.556577 (PMC7667464; doi:10.3389/fpsyg.2020.556577)
Supplement: Supplementary file 1 [file Table_1.DOCX]

# Appendix 1

SEARCH, STRUCTURAL

PubMed:

| HORMONAL CONTRACEPTIVES | STRUCTURAL BRAIN IMAGING STUDIES |
| --- | --- |
| “contraceptive agents”[mh] OR contracepti*[tiab] OR "birth control"[tiab] | “tomography, x-ray computed”[mh] OR computed axial tomography[tiab] OR cat[tiab] OR computed tomography[tiab] OR ct[tiab] OR “magnetic resonance imaging”[mh] OR mri[tiab] OR “diffusion tensor imaging”[mh] OR dti[tiab] OR “diffusion magnetic resonance imaging”[mh] OR diffusion weighted imaging[tiab] OR high angular resolution diffusion imaging[tiab] OR hardi[tiab] OR “image cytometry”[mh] OR morphometr*[tiab] OR voxel based morphometry[tiab] OR vbm[tiab] OR structural neuroimaging[tiab] OR cortical thickness[tiab] OR “gray matter”[mh] OR gr?y matter[tiab] |

Embase (OVIDSP):

| HORMONAL CONTRACEPTIVES | STRUCTURAL BRAIN IMAGING STUDIES |
| --- | --- |
| contracepti* OR birth control | computer assisted tomography OR CT OR DTI OR diffusion tensor imaging OR high angular resolution diffusion imaging OR diffusion weighted imaging OR structural neuroimaging OR scintigraphy OR cortical thickness OR gray matter OR brain |

PsycINFO (OVIDSP):

| HORMONAL CONTRACEPTIVES | STRUCTURAL BRAIN IMAGING STUDIES |
| --- | --- |
| contracepti* OR birth control | computer assisted tomography OR CT OR DTI OR diffusion tensor imaging OR high angular resolution diffusion imaging OR diffusion weighted imaging OR structural neuroimaging OR scintigraphy OR cortical thickness OR gray matter OR brain |

# Appendix 2

SEARCH, FUNCTIONAL:

Pubmed:

| HORMONAL CONTRACEPTIVES | FUNCTIONAL BRAIN IMAGING STUDIES |
| --- | --- |
| “contraceptive agents”[mh] OR contracepti*[tiab] OR birth control[tiab] | fMRI[tiab] OR functional magnetic[tiab] OR “positron-emission tomography”[mh] OR “positron emission tomography computed tomography”[mh] OR PET[tiab] OR positron emission tomography[tiab] OR “tomography, emission computed, single photon”[mh] OR SPECT[tiab] OR single photon emission computed tomography[tiab] OR “electroencephalography”[mh] OR EEG[tiab] OR electroencephalogra*[tiab] OR “magnetoencephalography”[mh] OR MEG[tiab] OR “spectroscopy, near-infrared”[mh] OR near infrared spectroscopy[tiab] OR “optical imaging”[mh] OR doi[tiab] OR diffuse optical imaging[tiab] OR diffuse optical tomography[tiab] OR dot[tiab] OR high density diffuse optical tomography[tiab] OR event related optical signal[tiab] OR EROS[tiab] OR “electrocorticography”[mh] OR ECOG [tiab] OR electrocorticogra*[tiab] OR ieeg[tiab] OR “evoked potentials”[mh] OR event related potential*[tiab] OR ERP[tiab] OR “evoked potentials, visual”[mh] OR VEP[tiab] OR “evoked potentials, auditory”[mh] OR auditory evoked potential*[tiab] OR AEP[tiab] |

Embase (OVIDSP):

| HORMONAL CONTRACEPTIVES | FUNCTIONAL BRAIN IMAGING STUDIES |
| --- | --- |
| contracepti* OR birth control | fMRI OR functional magnetic resonance imaging OR positron emission tomography OR SPECT OR single photon emission computed tomography OR electroencephalogra* OR magnetoencephalogra* OR near infrared spectroscopy OR diffuse optical imaging OR diffuse optical tomography OR high density diffuse optical tomography OR event related optical signal OR electrocorticogra* OR event related potential OR visual evoked potential OR auditory evoked potential |

PsycINFO (OVIDSP):

| HORMONAL CONTRACEPTIVES | FUNCTIONAL BRAIN IMAGING STUDIES |
| --- | --- |
| contracepti* OR birth control | fMRI OR functional magnetic resonance imaging OR positron emission tomography OR SPECT OR single photon emission computed tomography OR electroencephalogra* OR magnetoencephalogra* OR near infrared spectroscopy OR diffuse optical imaging OR diffuse optical tomography OR high density diffuse optical tomography OR event related optical signal OR electrocorticogra* OR event related potential OR visual evoked potential OR auditory evoked potential |
