# Supplementary material for: The Effects of Hormonal Contraceptives on the Brain: A Systematic Review of Neuroimaging Studies
Source: Front Psychol. 2020 Oct 27;11:556577. doi: 10.3389/fpsyg.2020.556577 (PMC7667464; doi:10.3389/fpsyg.2020.556577)
Supplement: Supplementary file 2 [file Table_2.DOCX]

| **Supplementary Table s1. Validity threats** | | | | |
| --- | --- | --- | --- | --- |
| **Structural studies** | | | | |
| **Study** | **Internal validity** | **External validity** | **Statistical conclusion validity** | **Construct validity** |
| De Bondt (2013a) ^37^ | Low: Participants self-selected into groups. | Low: Different HC with different androgenic properties used. Unknown duration of HC use. | Low: Sample size small and statistical power not calculated. | High: DTI is a validated measure of brain microstructure |
| De Bondt (2013b)^36^ | Low: Self-selected into the groups. | Low: Different HC with different androgenic properties used. Unknown duration of HC use. | Low: Sample size small and statistical power not calculated. | High: Voxel based morphometry is a validated measure of regional grey matter volume |
| De Bondt (2015)^30^ | Low: Self-selected into the groups. | Low: Different HC with different androgenic properties used. Unknown duration of HC use. | Low: Low rater agreement on quality assessment of spectra. Sample size small and statistical power not calculated | Low: Spectrograms of doubtful quality due to a too low number of averages and frequency drift of scanner. Validity of the measures in doubt |
| De Bondt (2016)^32^ | Low: Participants self-selected into the groups. Unbalanced attrition from the groups. | Low: Different HC with different androgenic properties used. Unknown duration of HC use. | Low: Sample size small and statistical power not calculated. | High: Voxel based morphometry validated by using second SPM package |
| Frokjaer (2009)^29^ | Low: Participants self-selected into the conditions. | Low: Different HC with different androgenic properties used. Unknown duration of HC use. | Low: Sample size small and statistical power not calculated. Study had negative findings, which are uninterpretable. | High: cortical 5-HT_2A_ receptor binding technique validated |
| Lisofsky (2016)^28^ | Intermediate: Patients self-selected start use of HCs, but study was a longitudinal within-between group effect study, reducing risk of bias. | Low: Different HC with different androgenic properties used. Unknown previous HC use prior to 6 months before study inclusion. | Intermediate: Sample size moderate, but in the context of positive findings and statistical power discussed. Risk of type 1 errors adjusted. | High: Well validated voxel based morphometry analyses. |
| Petersen (2015)^80^ | Low: Self-selection into groups. | Low: Undisclosed HC used. Undisclosed previous HC use. | Intermediate: Sample size moderate. Reasons for ROI choice not clearly stated. Results with corrections for familywise error presented | High: Freesurfer cortical thickness approach is well described and validated. |
| Pletzer (2010)^31^ | Low: Self-selection into groups. | Low: Undisclosed HC used. Undisclosed HC usage history. | Low: Small sample size. Statistical power not discussed or assessed. Voxel-vise threshold was uncorrected. | Moderate: Old SPM5 version of SPM was known to have reliability problems but the procedure is well validated. |
| Pletzer (2015)^29^ | Low: Self-selection into groups, also at an ad-hoc follow-up examination. | Moderate: Type of HC accounted for and history of HC use reported. Other aspects of sample not described in detail. | Low: Very small sample size when accounting for type of HC. Statistical power not discussed or assessed. | Moderate: Old SPM5 version of SPM was known to have reliability problems but the procedure is well validated. |
| Pletzer (2019)^63^ | Low: Self-selection into groups. | Low: Not all women reported HC-use and type of HC. Mixed HC use and prior use. | Intermediate: Moderate sample size. Statistical power not assessed or discussed. | High: The volumetric analyses were performed with well validated tools (SPM12). |
| **Functional studies** | | | | |
| **Study** | **Internal validity** | **External validity** | **Statistical conclusion validity** | **Construct validity** |
| Abler (2013)^47^ | Low: Self-selection into the groups. | Low: Type of HC were mixed, and duration of previous use was unaccounted for. Sample sparsely described. | Low: Small sample size, uncorrected voxel thresholds and no discussion or assessment of statistical power. | Low: The sexual film stimuli may differ from “neutral” stimuli in unknown ways. Procedure not validated in the fMRI context. Hence, what was measured is not clear. Older SPM5 used for analyses. |
| Arnoni-Bauer (2017)^50^ | Low: Self-selection into groups. | Intermediate: Same HC compounds used in all HC subjects. Sample very sparsely described. | Low: Small sample size. Power analysis done, accepting a power of 80%, but incomplete regarding all comparisons and effects of Bonferroni corrections. | High: fMRI stimulus paradigm well designed with regard to confounders. Validated fMRI analysis tool (Brain voyager). |
| Basu (2016)^51^ | Low: Self-selected subjects and pre-post quasi-experimental design without control group. | Intermediate: All subjects received same HC compound. Sample sparsely described. Previous use not described. | Low: Very small sample (n=8). | High: fMRI stimulus paradigm well designed with regard to confounders. Validated fMRI analysis tool. |
| Bonenberger (2013)^48^ | Low: Self-selection into the groups. | Low: Type of HC were mixed, and duration of previous use was unaccounted for. Sample sparsely described. | Low: Small sample size. Uncorrected voxel thresholds and no discussion or assessment of statistical power. | High: Validated fMRI task and fMRI analysis approach. |
| Chung (2016)^46^ | Low: Self-selection into the groups. | Low: Mixed HC types and lack of reported detail. | Low: Small sample size and no discussion or assessment of statistical power. | High: A validated fMRI social stress task was used. SPM8 validated for the fMRI analyses. |
| De Bondt (2015)^60^ | Low: Self-selection into groups. Different number of fMRI examinations in the groups. Missing data from groups non-random. | Low: Mixed HC types and lack of reported detail. Lack of sample information. | Low: Small sample size | Intermediate: Resting state fMRI subject to disagreement regarding construct validity. |
| Gingnell (2013)^39^ | High: Randomized controlled trial, with a sample of women with previous mood symptoms from HC use. Negligible attrition. | High: All HC women received same compound, and all had previous use. Sample and recruitment well described. | Intermediate: Power analysis indicated 94% statistical power, but sample size small and subgroup analyses probably underpowered. | High: Validated fMRI paradigm and validated psychometric tools used. |
| Gingnell (2016)^58^ | High: Randomized controlled trial, with a sample of women with previous mood symptoms from HC use. Negligible attrition. | High: All HC women received same compound, and all had previous use. Sample and recruitment well described. | Low: Small sample size and no assessment or discussion about statistical power. | Intermediate: Go/NoGo task not referenced, hence being generic with unknown construct validity regarding “cognitive inhibition” in an fMRI setting. |
| Hornung (2019)^40^ | Low: Self-selection into groups. | Low: Mixed and undescribed HC used and undisclosed previous use. | Intermediate: A-priori power analysis yielding 80% power, but not clear to what degree the analyses were relevant for the fMRI analyses. Corrections for multiple comparisons were performed. | Intermediate: Dot-probe task not clear with regard to interpretation but based on previous studies. |
| Hwang (2015)^44^ | Low: Self-selected subjects into the groups. | Low: Undisclosed HC types or previous use. | Low: Sample size small. No discussion or assessment of statistical power. | High: Fear conditioning a validated and well described construct. |
| Lisofsky (2016)^32^ | Intermediate: Patients self-selected start use of HCs, but study was a longitudinal within-between group effect study, reducing risk of bias. | Low: Different HC with different androgenic properties used. Unknown previous HC use prior to 6 months before study inclusion. | Low: Sample size moderate. fMRI analyses were unadjusted with regard to familywise error. | Intermediate: Resting state fMRI subject to disagreement regarding construct validity. |
| Marečková (2014)^54^ | Low: Self-selection into groups. | Low: Different HC and lack of detail regarding sample properties. | Low: Small sample size and statistical power not assessed or discussed. | Intermediate: Previously published fMRI paradigm, but interpretation of the bold responses not clear. |
| Merz (2012)^41^ | Low: Self-selection into HC group. | Low: Different HC used and lack of information regarding previous use. | Intermediate: Sample size moderate. fMRI analyses corrected for familywise error risk. Statistical power not assessed or discussed. | High: Fear learning paradigm well established and validated previously. |
| Merz (2013)^42^ | Low: Self-selection into groups. | Low: Different HC used and lack of information regarding previous use. | Low: Small sample size. Statistical power not discussed or assessed. | High: Fear learning paradigm well established and validated previously. |
| Miedl (2018)^45^ | Low: Self-selection into groups. | Low: Different HC used and lack of information regarding previous use. Sample sparsely described. | Intermediate: Sample size moderate but statistical power not assessed or discussed. Familywise error correction done. | Low: Not clear how or if this fMRI paradigm relates to real trauma or post-traumatic stress. |
| Monciunskaite (2019)^38^ | Low: Self-selection into groups | Intermediate: Different HC used, but brands reported, and all were anti-androgenic. Previous use not disclosed. | Intermediate: Sample size moderate but statistical power not assessed or discussed. | Intermediate: Validated stimulus material but not clear how the ERP-measures relate to the assessed construct. |
| Petersen (2014)^59^ | Low: Self-selection into groups | Low: HC type details not reported and no information on previous use. | Intermediate: Moderate sample size but no discussion or assessment of statistical power | Intermediate: ICA approach appears very subjective, but inter-rater agreement not discussed. |
| Petersen (2015)^43^ | Low: Self-selection into groups | Low: Mixed HC type details reported but no information on previous use. | Intermediate: Moderate sample size but no discussion or assessment of statistical power | High: Validated stimulus material and relevant region of interest analyses. |
| Pletzer (2014)^56^ | Low: Self-selection into groups. | Low: HC type not reported. Previous use not disclosed. | Low: Small sample size. Statistical power not assessed or discussed. Familywise error not adjusted for in primary thresholds. | Intermediate: Tasks were previously validated by the same group, but the constructs were elaborate and not clearly conveyed. |
| Rumberg (2010)^57^ | Low: Self-selection into groups. | Low: Undisclosed HC used. No information about previous use. | Low: Small sample size. Statistical power not assessed or discussed. Analyses not corrected for familywise error. | Low: Verb generation task not described well and not referenced. |
| Scheele (2016)^49^ | Low: Self-selection into groups. | Low: Undisclosed HC used. No information about previous use. | Low: Sample size small for subjects with valid data. Statistical power not assessed or discussed. | Intermediate: Face perception task previously validated but relation to experimental design not straightforward regarding interpretation. |
| Vincent (2013)^53^ | Low: Self-selection into groups. | Low: Type of HC not disclosed. Previous use not disclosed. | Low: Small sample size. Statistical power not assessed. | Intermediate: Pain paradigm validated behaviorally, but not with regard to fMRI. |
| Validity typology based on the framework described in Cook and Campbell(27)  Sample size is classified as “small” if number of subjects in the HC and comparison group was N<20. | | | | |
